# Shared roles of immune and stromal cells in the pathogenesis of human bronchiolitis obliterans syndrome

**DOI:** 10.1172/jci.insight.176596

**Published:** 2025-04-15

**Authors:** Patrick W. Mellors, Ana N. Lange, Bruno Casino Remondo, Maksim Shestov, Joseph D. Planer, Andrew R. Peterson, Yun Ying, Su Zhou, Jason D. Christie, Joshua M. Diamond, Edward Cantu, Maria C. Basil, Saar Gill

**Affiliations:** 1 Division of Hematology/Oncology, Department of Medicine, University of Pennsylvania (Penn), Philadelphia, Pennsylvania, USA.; 2 Center for Cellular Immunotherapies and; 3 Department of Medicine, Perelman School of Medicine, Penn, Philadelphia, Pennsylvania, USA.; 4 Penn-CHOP Lung Biology Institute and; 5 Penn Cardiovascular Institute, Perelman School of Medicine, Philadelphia, Pennsylvania, USA.; 6 Department of Surgery, Perelman School of Medicine, Penn, Philadelphia, Pennsylvania, USA.

**Keywords:** Hematology, Immunology, Pulmonology, Bioinformatics, Bone marrow transplantation, Organ transplantation

## Abstract

Bronchiolitis obliterans syndrome (BOS) is a progressive, fatal obstructive lung disease that occurs following lung transplant, where it is termed chronic lung allograft dysfunction BOS (CLAD-BOS), or as the primary manifestation of pulmonary chronic graft versus host disease (cGVHD-BOS) following allogeneic hematopoietic stem cell transplant. Disease pathogenesis is poorly understood; however, chronic alloreactivity is common to both conditions, suggesting a shared pathophysiology. We performed single-cell RNA-Seq (scRNA-Seq) on explanted human lungs from 4 patients with CLAD-BOS, 3 patients with cGVHD-BOS, and 3 deceased controls to identify cell types, genes, and pathways enriched in BOS to better understand disease mechanisms. In both forms of BOS, we found an expanded population of CD8^+^ tissue resident memory T cells (TRM), which was distinct to BOS compared with other chronic lung diseases. In addition, BOS samples expressed genes and pathways associated with macrophage chemotaxis and proliferation, including in nonimmune cell populations. We also identified dysfunctional stromal cells in BOS, characterized by pro- and antifibrotic gene programs. These data suggest substantial cellular and molecular overlap between CLAD- and cGVHD-BOS and, therefore, common pathways for possible therapeutic intervention.

## Abstract 2

Obliterative bronchiolitis after lung or hematopoietic stem cell transplantation is characterized by an expansion of activated tissue resident memory T cells, macrophage chemotaxis and proliferation, and dysfunctional stromal cells.

## Graphical abstract

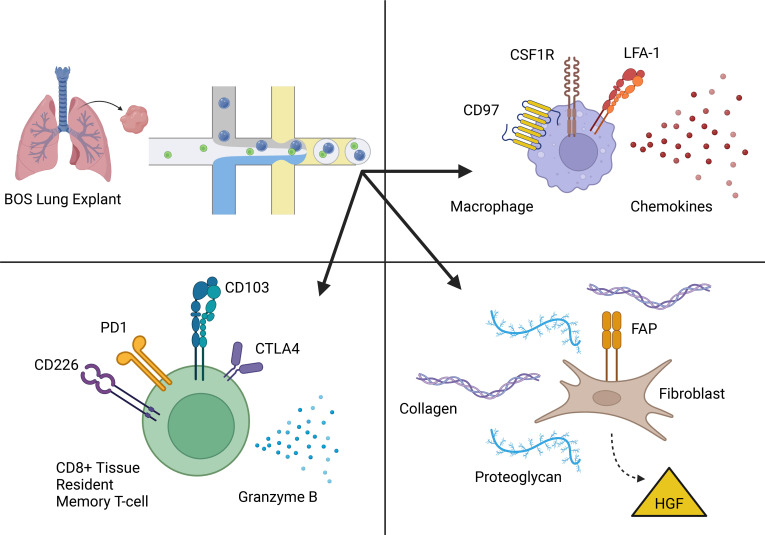

## Introduction

Bronchiolitis obliterans syndrome (BOS) is an obstructive lung disease in which fibrotic obliteration of the small airways leads to progressive respiratory failure. BOS occurs as a complication of lung transplant, where it is termed chronic lung allograft dysfunction BOS (CLAD-BOS), or as the primary manifestation of pulmonary chronic graft versus host disease (cGVHD-BOS) following allogeneic hematopoietic stem cell transplant (ASCT). CLAD-BOS occurs in 50% of surviving lung transplant recipients at 5 years and is the leading cause of late lung allograft failure (1). In patients undergoing ASCT, 5%–10% develop cGVHD-BOS, and prognosis is poor, with only half of patients surviving 5 years after ASCT (2). At best, current treatments produce modest improvement in airway obstruction for a minority of patients, predominantly with early-stage disease (3, 4). Fibrosis or pathologic airway remodeling is not currently thought to be reversible (4–7). Patients with late-stage disease have few treatment options other than lung transplantation.

Data from human peripheral blood and BAL samples implicate the innate immune response in CLAD-BOS pathogenesis (8–10). Adaptive cellular immunity and antibody responses are also involved, and acute rejection and donor-specific antibodies increase CLAD-BOS risk (11–15). Airway remodeling may be driven by aberrant wound healing and tissue fibrosis, mediated by TGF-β and activated, dysfunctional fibroblasts (1, 16, 17). Although no single murine model fully replicates all clinical features of human cGVHD, current models inform much of our current understanding of cGVHD-BOS pathogenesis (18). For example, Th17-dependent, CSF1R-expressing macrophages in pulmonary tissues are pathogenic, and CSF1R blockade abrogates BOS development (19). Allo-antibody production in mice is also associated with fibrogenesis (20), and Th17 cells are necessary for the development of cGVHD-BOS in murine models (21).

Despite these findings, the pathogenesis of CLAD- and cGVHD-BOS remains poorly understood. This knowledge gap is likely attributable to a paucity of animal models and limited data from primary human tissue. Murine models of BOS (19) are limited by species difference between mice and humans, particularly by the absence of respiratory bronchioles (RB) in murine lungs, which are the anatomic site of disease in human BOS. Available in-human analyses focus on changes in peripheral blood (22) or bronchoalveolar lavage (BAL) fluid (23, 24). However, unlike tissue, BAL samples cannot capture parenchymal/interstitial cell populations or provide spatial resolution. A recent study addressed these limitations through single-cell RNA-Seq (scRNA-Seq) of explanted lung tissue from patients with CLAD-BOS (25).

To interrogate relationships between tissue-resident immune and nonimmune cells within the lung parenchyma, we performed scRNA-Seq of lungs from patients with CLAD- or cGVHD-BOS. We identify and characterize a subset of CD8^+^ T cells with a tissue resident memory (TRM) phenotype in the lungs of patients with BOS that are specific to this lung disease. We confirm TRM expansion in BOS using multiplex immunofluorescence (mIF) and illuminate their spatial distribution around remnant airways and in densely fibrotic lung parenchyma. Our data also highlight the role of macrophage chemotaxis and proliferation in BOS pathogenesis. We also identify stromal cells in BOS with pro- and antifibrotic gene expression profiles. Identification of these TRM, macrophage, and stromal signatures in 4 patients with CLAD-BOS and 3 patients with cGVHD-BOS but not in healthy controls suggests a common pathogenesis and potentially, shared avenues for therapeutic intervention.

## Results

### Cell clustering and identification in CLAD-BOS.

**Figure 1:**
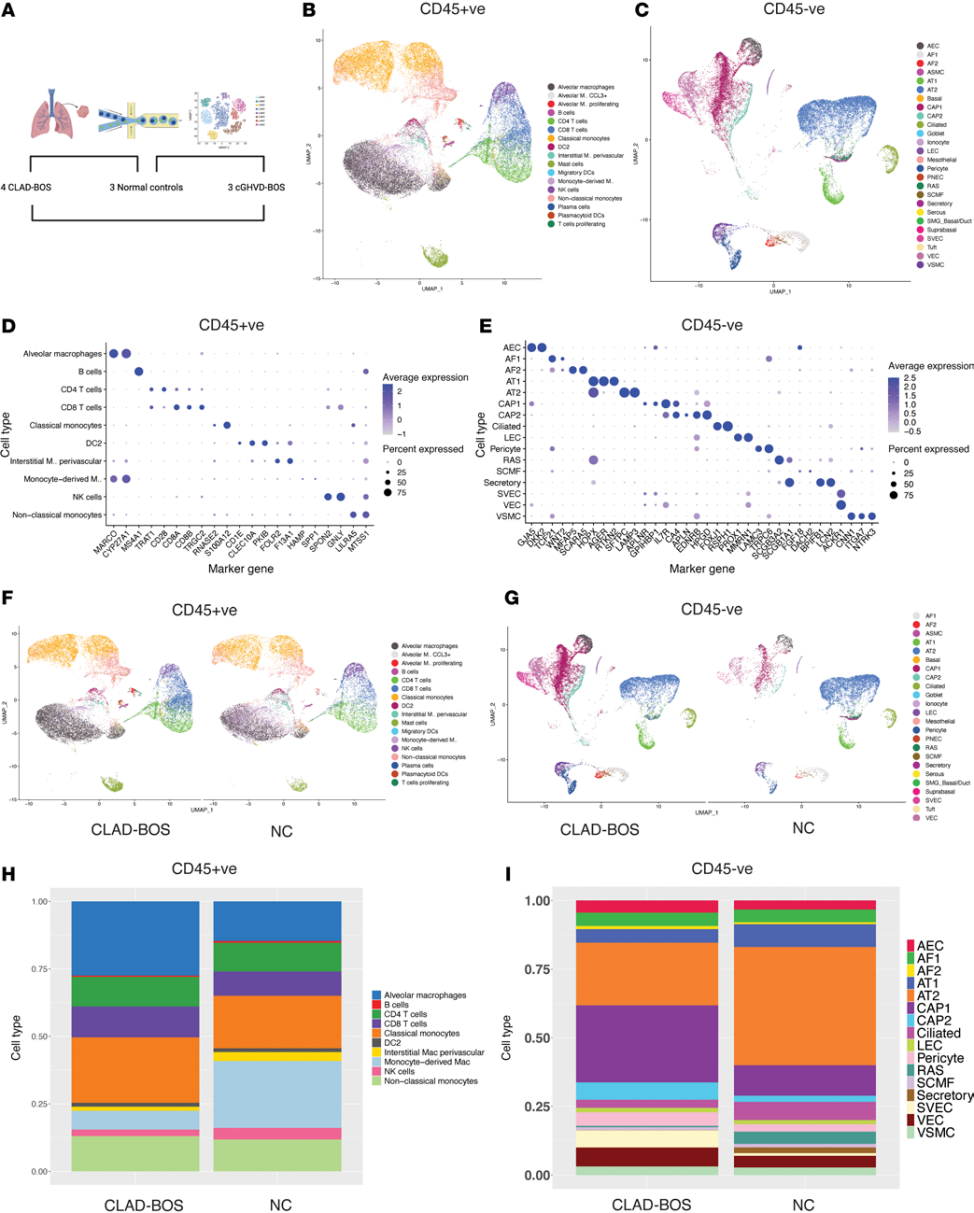
Single-cell landscape of chronic lung allograft dysfunction associated bronchiolitis obliterans syndrome (CLAD-BOS). (**A**) Schematic of overall study design, with brackets indicating planned comparisons between CLAD-BOS and normal control (NC) lungs, cGVHD-BOS and NC lungs, and CLAD- and cGVHD-BOS lungs. (**B** and **C**) UMAP projections illustrating CD45^+^ and CD45^–^ cell populations in CLAD-BOS and NC. (**D** and **E**) Dot plot illustrating marker genes for CD45^+^ and CD45^–^ cell types with > 300 cells from CLAD-BOS and NC. (**F** and **G**) UMAP projections for CD45^+^ and CD45^–^ cell types stratified by condition. (**H** and **I**) Bar plots with cell type fractions for CD45^+^ and CD45^–^ cell types with > 300 cells stratified by condition. AEC, arterial endothelial cells; AF1, type 1 alveolar fibroblasts; AF2, type 2 alveolar fibroblasts; ASMC, airway smooth muscle cells; AT1 type 1, alveolar epithelial cells; AT2, type 2 alveolar epithelial cells; Cap1, type 1 capillary cells; Cap2, type 2 capillary cells; LEC, lymphatic endothelial cells; PNEC, pulmonary neuroendocrine cells; RAS, respiratory airway secretory cells; SCMF, secondary crest myofibroblasts; SMG_Basal/Duct, submucosal gland basal/duct cells; SVEC, systemic venous endothelial cells; VEC, venous endothelial cells; VSMC, venous smooth muscle cells.

To interrogate the cellular environment of CLAD-BOS, we performed scRNA-Seq on enriched immune (CD45^+^) and nonimmune (CD45^–^) fractions from distal lung explants from patients undergoing second lung transplantation with CLAD-BOS (*n* = 4) compared with normal age- and sex-matched controls (NC) (*n* = 3) (Figure 1A and Supplemental Table 1; supplemental material available online with this article; https://doi.org/10.1172/jci.insight.176596DS1). After quality control filtering, we identified 40,067 CD45^+^ cells (22,248 in CLAD-BOS and 17,819 in NC) and 30,027 CD45^–^ cells (19,862 in CLAD-BOS and 10,165 in NC). Using reference mapping (26, 27), we identified 17 distinct cell types in the CD45^+^ fraction (Figure 1B) and 26 in the CD45^–^ fraction (Figure 1C). With some exceptions in rare cell populations, most cells had high confidence annotations defined as prediction scores > 0.75 (Supplemental Figure 1, A and B). We validated these reference-based cell type predictions using canonical marker genes (Figure 1, D and E, and Supplemental Figure 1, C and D). CD45^+^ and CD45^–^ UMAP projections (Figure 1, F and G) and cell type fractions (Figure 1, H and I), stratified by condition, suggest expansion of alveolar macrophages (aMac), CD8^+^ T cells, stromal and endothelial cell subtypes, and contraction of epithelial cell populations in CLAD-BOS. Thus, compared with NC, CLAD-BOS lungs had a more robust cytotoxic T cell and aMac infiltrate and evidence of expanded stromal and endothelial cell subsets.

### TRM T cells are identified in CLAD-BOS.

**Figure 2:**
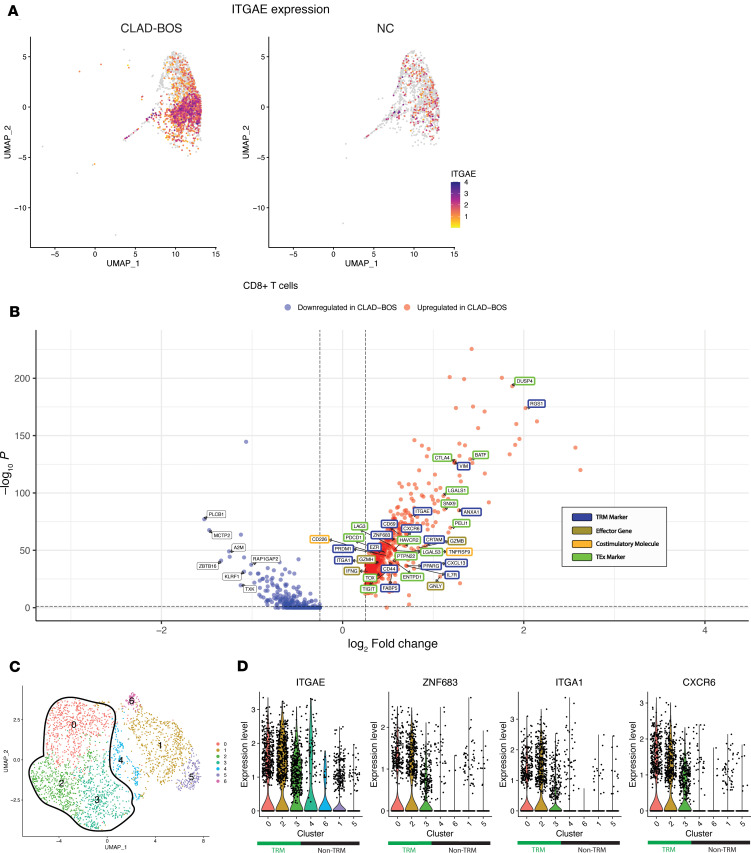
CD8^+^ T cells with a tissue resident memory (TRM) phenotype are enriched in CLAD-BOS. (**A**) Feature plot with *ITGAE* (encoding CD103) expression in CD8^+^ T cells in patients with CLAD-BOS versus NC lungs. (**B**) Volcano plot illustrating upregulation of genes for (a) canonical TRM markers, (b) effector function proteins, (c) costimulatory molecules, and (d) inhibitory receptors and transcription factors associated with T cell exhaustion in CD8^+^ T cells from CLAD-BOS compared with NC. Differences in gene expression were quantified using log_2_ fold change and statistically significant differences were identified using the Wilcoxon Rank Sum Test and a Bonferroni-corrected *P* < 0.05. (**C**) UMAP projection of reclustered CD8^+^ T cells from 4 CLAD-BOS lungs grouped by cluster. (**D**) Violin plot with expression of canonical TRM marker genes in CLAD-BOS by cluster. TRM clusters are highlighted in green on the *x* axis.

We observed that 49% of CD8^+^ T cells in patients with CLAD-BOS expressed the TRM marker *ITGAE* (encoding CD103) compared with 16% in NC, suggesting that TRM were heavily enriched in CLAD-BOS (Figure 2A and Supplemental Figure 2A). Differential gene expression analysis of CD8^+^ T cells demonstrated upregulation of the TRM markers *ITGAE*, *CD69*, and *ITGA1* (encoding CD49a) in CLAD-BOS (Figure 2B and Supplemental Table 2) (28, 29). Upregulated genes also included *VIM* (30, 31), *EZR* (30, 31), *CD44* (32), *CRTAM* (33), and *ANXA1* (31), which encode adhesion, cytoskeletal, and scaffolding proteins shown to facilitate TRM tissue retention (Figure 2B). *RGS1*, which prevents TRM tissue egress and lymph node homing, and the TRM-associated chemokine *CXCL13* and chemokine receptor *CXCR6* were also enriched in CD8^+^ T cells in patients with CLAD-BOS (Figure 2B) (30, 33–36). *IL7R*, which is frequently identified in TRM (36, 37) and promotes T cell survival and persistence in tissues (38, 39), was also highly expressed (Figure 2B). Likewise, the TRM-associated transcription factors *PRDM1* (encoding BLIMP-1) and *ZNF683* (encoding Hobit) were upregulated (Figure 2B) (40). Lastly, TRM rely on fatty acid metabolism, facilitated by the gene *FABP5* and its upstream regulator, *PPARG*, to survive and persist in nutrient-deficient barrier sites (41), and these genes were enriched in CD8^+^ T cells in patients with CLAD-BOS (Figure 2B).

Genes associated with T cell exhaustion (TEx) and those encoding effector function proteins are frequently coexpressed in TRM (30, 33, 35, 36, 42–45). Indeed, CD8^+^ T cells in patients with CLAD-BOS mirrored this archetypal TRM transcriptional signature. Multiple inhibitory receptors (IR) and transcription factors associated with TEx (46–48) were upregulated in CD8^+^ T cells in patients with CLAD-BOS (Figure 2B). *DUSP4*, which is implicated in T cell senescence (49); *SNX9*, a newly described mediator of TEx (50); and T cell–suppressive genes, including *LGALS1*, *LGALS3*, and *PELI1*, were also upregulated (51, 52).

IR upregulation in CLAD-BOS CD8^+^ subsets was concomitant with that of effector protein genes *GZMB*, *GZMH*, *IFNG*, and *GNLY* (Figure 2B), which have functional roles in CD8^+^ T cell cytotoxicity. In addition, we found evidence for T cell activating programs in CLAD-BOS. Costimulatory molecule genes associated with T cell activation including *CD226* (53), *TNFRSF9* (encoding 4-1BB) (54), and *CRTAM* (which may also promote TRM tissue adhesion and retention) (33, 55) were highly expressed in CD8^+^ T cells in patients with CLAD-BOS (Figure 2B).

We next performed differential gene expression on the subset of ITGAE^+^ cells to explore if the patterns of upregulation in CLAD-BOS CD8^+^ T cells were driven by a larger quantity of TRM in CLAD-BOS or by qualitative differences in TRM between conditions. ITGAE^+^ cytotoxic T cells in patients with CLAD-BOS had higher expression of genes associated with TEx, effector function, and T cell stimulation and activation compared with those in NC (Supplemental Figure 2B and Supplemental Table 3). These findings indicate qualitative and not just quantitative differences in CD8^+^ TRM cells in patients with CLAD-BOS compared with NC.

**Figure 3:**
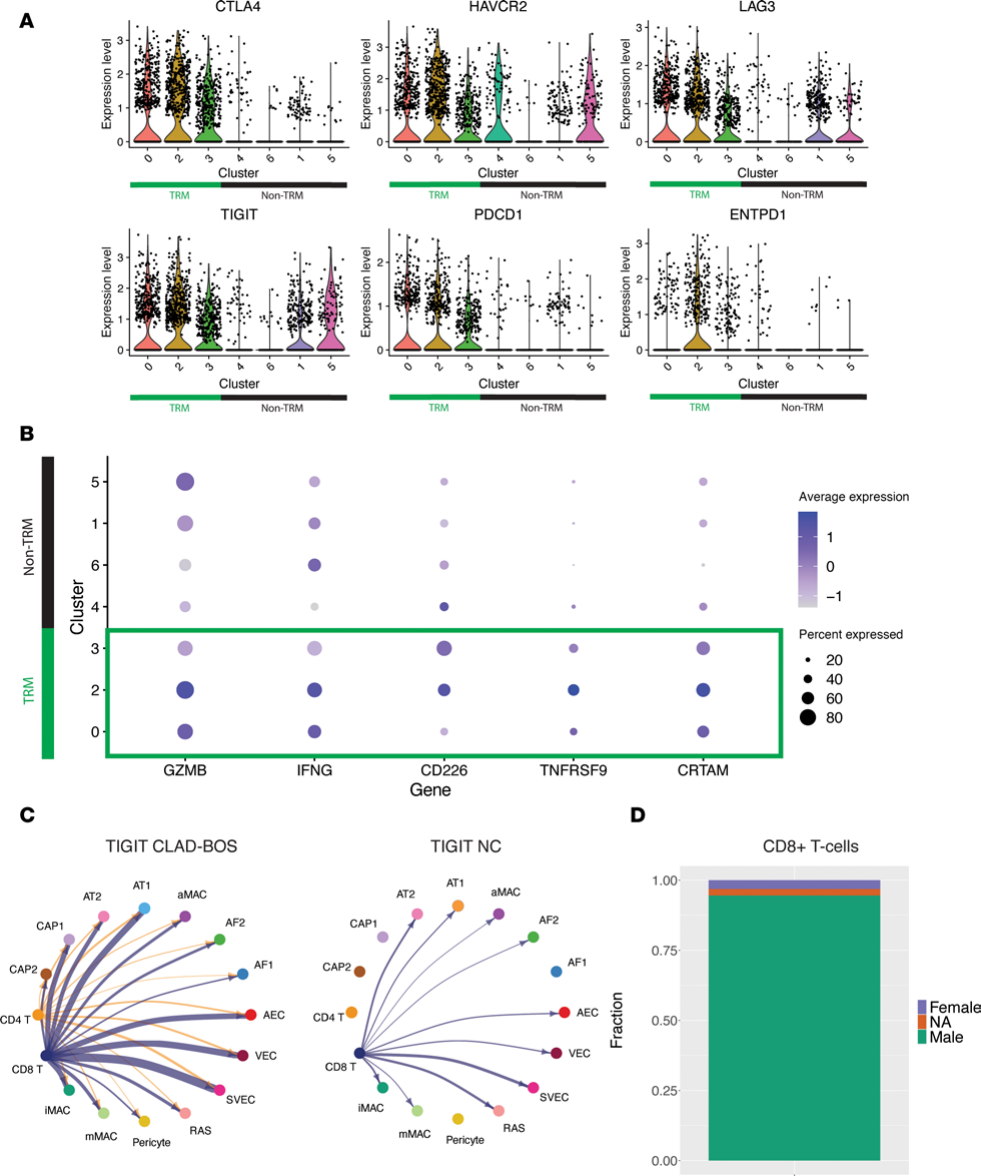
TRM express inhibitory receptor, effector function protein, and costimulatory molecule genes and are primarily recipient derived in CLAD-BOS. (**A**) Violin plots illustrating cluster-based expression of inhibitory receptors in CD8^+^ T cells in patients with CLAD-BOS, with TRM clusters highlighted with green. (**B**) Dot plot showing scaled expression of effector function protein and T cell costimulatory molecule genes by cluster, with TRM clusters outlined in green. (**C**) *TIGIT* signaling in CLAD-BOS compared with NC. Lines connect interacting cells (dots), and cells sending outgoing signal have the same color as the corresponding line. All interactions depicted are statistically significant using a 1-sided permutation test with significance threshold of *P* < 0.05, and thicker lines indicate stronger interactions. (**D**) Sex of lung-derived CD8^+^ T cells in a male patient with CLAD-BOS with a female lung donor assigned using the speckle package, which employs logistic regression and multiple X- and Y-associated genes to predict cell sex. NA signifies that cell sex could not be determined due to lack of expression of X and Y associated genes.

We reclustered CLAD-BOS CD8^+^ T cells to explore coexpression of tissue residency markers with TEx, effector, and T cell activating/costimulatory genes (Figure 2C). This revealed a consistent concentration of the TRM genes *ITGAE*, *ZNF683*, *ITGA1*, and *CXCR6* in clusters 0, 2, and 3 (TRM clusters) (Figure 2D). TRM clusters expressed genes encoding IR (Figure 3A), T cell costimulatory molecules (Figure 3B), and effector function proteins (Figure 3B). Density plots for these genes of interest support their coexpression within individual cells (Supplemental Figure 3, A–G).

Non-TRM clusters 6, 1, 5, and 4 (Figure 2C) were consistent with circulating, terminally differentiated effector memory T cells (T-TEM). These clusters expressed genes encoding T-TEM surface molecules (56, 57), proteins promoting tissue egress and lymph node homing (33), effector function proteins like granzyme B and perforin, and transcription factors associated with terminal differentiation including T-bet (*TBX21*) and *ZEB2* (56, 57) (Supplemental Figure 2C).

In contrast to CLAD-BOS, reclustering of NC (Supplemental Figure 4A) did not reveal distinct TRM clusters (Supplemental Figure 4B). While cluster 2 expressed *ITGAE* and *ITGA1*, IR expression was low in this cluster (Supplemental Figure 4C). T cell costimulatory and effector function genes were weakly expressed in cluster 2 (Supplemental Figure 4D). Most clusters (clusters 0, 1, 3, and 5) in NC were consistent with circulating T-TEM with a similar phenotype to that identified in CLAD-BOS (Supplemental Figure 4E). Taken together, this suggests that CD8^+^ T cells in patients with CLAD-BOS include a discrete population of TRM bearing both IR, effector and costimulatory genes that are not identified in NC.

We further explored coexpression of T cell activating and suppressing molecules in cell-cell/ligand-receptor interaction modeling. T cell immunoreceptor with immunoglobulin and ITIM domain (*TIGIT*) and *CD226* inhibit and activate T cells, respectively, are frequently coexpressed on the same cell, and they compete for binding of human poliovirus receptor CD155(*PVR*) and CD112 (*Nectin-2*) on antigen presenting cells (58). *TIGIT* has been previously identified in TRM (42, 43, 59, 60), but data on *CD226* coexpression is sparse. We observed possible signaling from CD8^+^ and CD4^+^ T cells expressing *TIGIT* to epithelial, endothelial, stromal, and macrophage subsets via *PVR* and *Nectin-2* (Figure 3C and Supplemental Figure 5A) in CLAD-BOS. Possible *CD226* signaling from CD8^+^ T cells occurred exclusively in CLAD-BOS and mirrored the pattern of *TIGIT* signaling (Supplemental Figure 5, B and C). This possible concurrent *TIGIT* and *CD226* signaling in CLAD-BOS suggests a dynamic balance between immune regulation and activation, which is frequently described in the physiology of TRM responses (30, 33, 35, 36, 42–45).

### Cytotoxic T cells in patients with CLAD-BOS are recipient derived.

Since donor antigen is expected to elicit an alloreactive immune response from recipient T cells in chronic rejection, we hypothesized that CD8^+^ T cells in patients with CLAD-BOS would primarily be of recipient origin. We used the differences in human leukocyte antigen (HLA) haplotype between donor and recipient to determine recipient or donor origin. As all CD45^–^ cells are of donor origin, type 2 alveolar epithelial cells (AT2) were typed as a control to confirm the accuracy of HLA predictions (Supplemental Table 4, A–D). Supporting our hypothesis, the HLA type of lung-derived CD8^+^ T cells matched that of the recipient (Supplemental Table 4, A–D). Next, we leveraged Y and X chromosome–associated gene expression in a male patient with CLAD-BOS who received lungs from a female donor to assign T cells a recipient or donor origin. As a control, we evaluated CD45^–^ cells from the 4 CLAD-BOS lung donors and 3 NC for expression of *XIST* and *RPS4Y1* as genes defining female and male origin, respectively (Supplemental Figure 6, A and B). As expected, the majority of lung-derived CD8^+^ T cells in the male recipient of a female lung graft were of male origin (Figure 3D), and results were similar for the ITGAE^+^ subset (Supplemental Figure 6C). In summary, CD8^+^ T cells in patients with CLAD-BOS were primarily of recipient origin.

### Macrophage migration, adhesion, and proliferation are enriched in CLAD-BOS.

**Figure 4:**
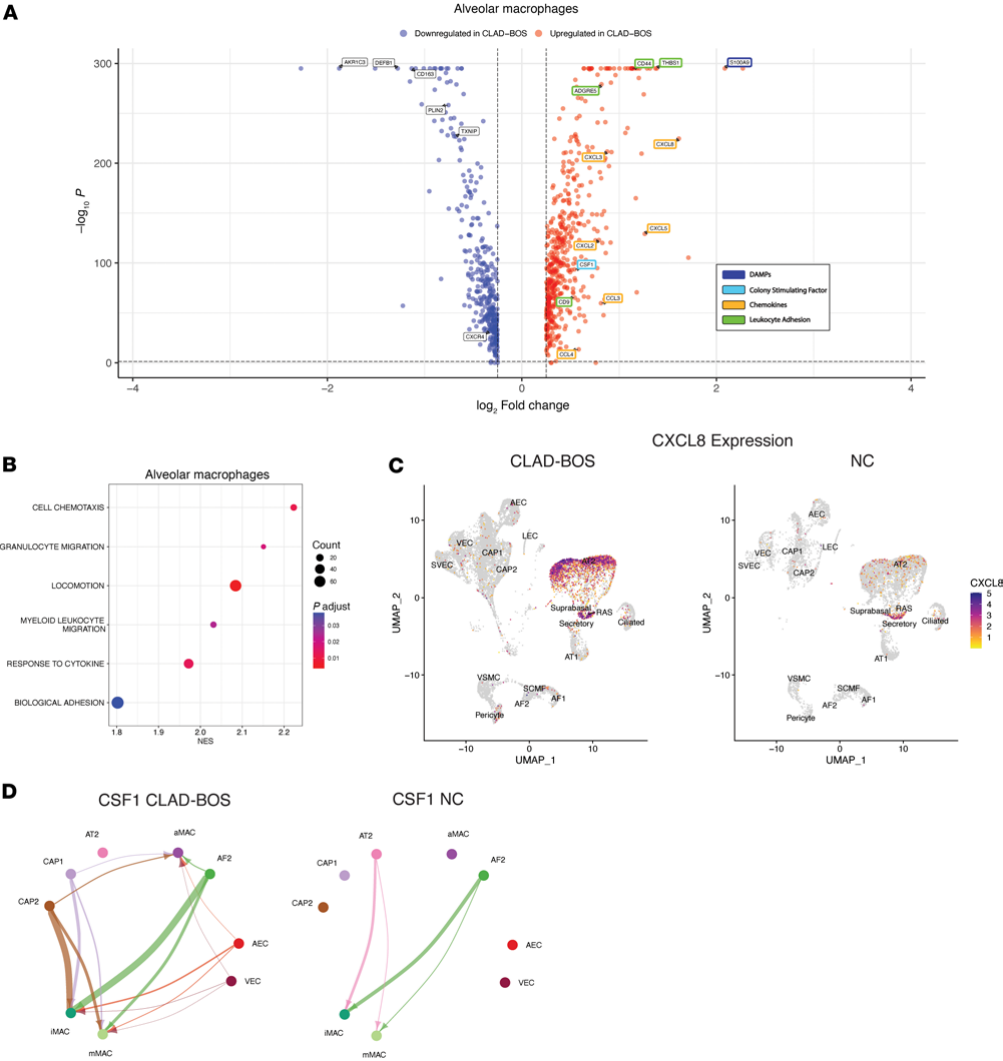
CLAD-BOS is enriched for genes, pathways, and cell-cell interactions promoting macrophage migration, adhesion, and proliferation. (**A**) Volcano plot illustrating differential expression of genes encoding (a) damage-associated molecular patterns (DAMPs), (b) colony stimulating factor, (c) chemokines, and (d) leukocyte adhesion proteins in alveolar macrophages in CLAD-BOS compared with NC lungs. Differences in gene expression were quantified using log_2_ fold change, and statistically significant differences were identified using the Wilcoxon rank sum test and a Bonferroni-corrected *P* < 0.05. (**B**) Dot plot with enrichment of gene ontology (GO) terms related to migration, adhesion, and cytokine response in alveolar macrophages in CLAD-BOS compared with NC. *P* values for normalized enrichment score (NES) were calculated using permutation testing and adjusted for multiple comparisons using the Benjamini-Hochberg method. (**C**) Feature plot illustrating *CXCL8* expression in CD45^–^ cell subsets in CLAD-BOS compared with NC. (**D**) Circle plot illustrating putative *CSF1* signaling from endothelial (CAP1, CAP2, AEC, VEC) and stromal cells (AF2) to macrophage subsets in CLAD-BOS versus NC. All interactions depicted are statistically significant using a 1-sided permutation test with significance threshold of *P* < 0.05, and thicker lines indicate stronger interactions.

Chemokines and adhesion proteins facilitate leukocyte migration to target organs in cGVHD and CLAD-BOS (24, 61). Multiple genes encoding chemokines and adhesion proteins were upregulated in aMac, interstitial perivascular macrophages (iMac), and monocyte-derived macrophages (mMac) in CLAD-BOS (Figure 4A; Supplemental Figure 7, A and B; and Supplemental Tables 5–7). Once in tissues, macrophage proliferation and differentiation in murine models of pulmonary cGVHD is mediated by CSF1-dependent CSF1R-expressing macrophages (19), and we observed upregulation of *CSF1* in aMac in CLAD-BOS (Figure 4A). Gene ontology terms for adhesion, macrophage migration/chemotaxis, and cytokine response were overrepresented in aMac (Figure 4B), again supporting a possible role for macrophage influx and function in BOS pathophysiology. Like CD8^+^ T cells, aMac were primarily recipient derived in patients with CLAD-BOS (Supplemental Table 4, A–D, and Supplemental Figure 7C).

In addition to immune cells, distal airway epithelial and stromal cells produce chemokines in inflammatory lung disease (62, 63), and we hypothesized that this would be true in CLAD-BOS. The monocyte/macrophage chemoattractant *CCL2* was among the most upregulated genes across CD45^–^ cell populations in CLAD-BOS compared with NCs (Supplemental Figure 7D). AT2 and respiratory airway secretory cells (RAS) were enriched for the chemokines CXCL8 (Figure 4C) and CXCL1 (Supplemental Figure 7E), which promote immune cell migration (64) and angiogenesis (65). Likewise, gene ontology terms related to adhesion, chemotaxis, and chemokine response were upregulated in AT2 and RAS cells (Supplemental Figure 7, F and G). Taken together, this supports the putative role of nonimmune cells in facilitating immune cell homing in CLAD-BOS.

We next explored possible interactions promoting macrophage entry into distal lung parenchyma. We identified stronger interactions between (a) aMac and mMac expressing *ITGB2* and *ICAM1* and *ICAM2* on endothelial cells (66) (Supplemental Figure 8, A–C) and (b) aMac and mMac expressing *ADGRE5* (encoding CD97) with *CD55* expressing epithelial, stromal, and endothelial cells (67, 68) (Supplemental Figure 8, D–G) in CLAD-BOS compared with NC. Possible interactions promoting macrophage proliferation and differentiation were also enriched in CLAD-BOS. Signaling from *CSF1* on endothelial cells to *CSF1R* on macrophage subsets was unique to CLAD-BOS (Figure 4D, Supplemental Figure 8, H–J). In summary, CLAD-BOS was enriched for genes, pathways, and possible cell-cell/ligand-receptor interactions, promoting macrophage recruitment to and proliferation within target lung tissue guided by epithelial, endothelial, and stromal parenchymal populations.

### Fibrosis and wound healing pathways are enriched in CLAD-BOS lungs.

**Figure 5:**
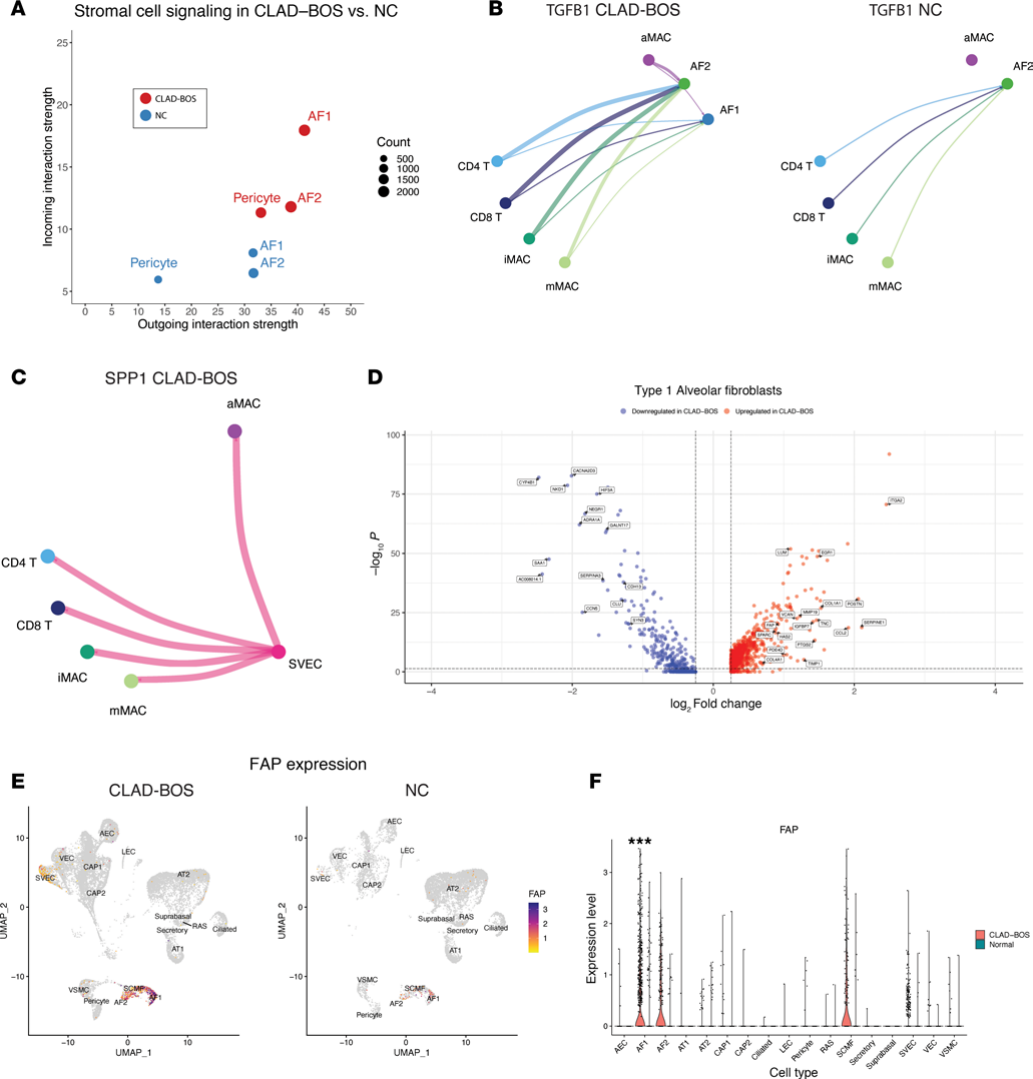
CLAD-BOS is enriched for pro- and antifibrotic genes and intercellular signaling. (**A**) Scatter plot of incoming (*y* axis) versus outgoing (*x* axis) signaling strength for stromal cells in patients with CLAD-BOS in red and NC in blue. (**B**) Circle plot illustrating interaction strength for *TGFB1* signaling in CLAD-BOS from macrophage and T cell populations to AF1 and AF2 compared with NC. All interactions depicted are statistically significant using a 1-sided permutation test with significance threshold of *P* < 0.05, and thicker lines indicate stronger interactions. (**C**) Circle plot showing interaction strength for putative osteopontin (*SPP1*) signaling in CLAD-BOS lungs from SVEC to T cell and macrophage populations (osteopontin signaling did not occur in NC). (**D**) Volcano plot illustrating differentially expressed pro- and antifibrotic genes for AF1 in CLAD-BOS compared with NC. Differences in gene expression were quantified using log_2_ fold change, and statistically significant differences were identified using the Wilcoxon rank sum test and a Bonferroni-corrected *P* value < 0.05. (**E**) Feature plot of *FAP* expression in CD45^–^ cell subsets in CLAD-BOS versus NC. (**F**) Violin plot showing *FAP* expression by CD45^–^ cell type, stratified by condition. ***P < 0.001 by Wilcoxon rank sum test, Bonferroni-corrected.

CLAD-BOS is defined histopathologically by fibrotic obliteration of RB, and tissue fibrosis is thought to be driven by stromal cells including fibroblasts and pericytes (69–71). We therefore modeled possible communications to and from stromal cells in patients with CLAD-BOS. Type 1 (AF1) and type 2 alveolar fibroblasts (AF2) and pericytes provided the strongest outgoing signaling in CLAD-BOS (Supplemental Figure 9A). Outgoing and incoming signaling strengths from AF1, AF2, and pericytes were also greater in CLAD-BOS compared with NCs (Figure 5A). Thus, stromal cells in patients with CLAD-BOS may serve as a hub for intercellular signaling, implicating them in disease pathogenesis.

A possible increase in profibrotic signaling was observed in CLAD-BOS. T cells and macrophages expressing *TGFB1*, a critical regulator of fibrosis, had stronger signaling to AF2 in CLAD-BOS, and signaling to AF1 only occurred in CLAD-BOS (Figure 5B and Supplemental Figure 9B). Systemic venous endothelial cells (SVEC) expressing osteopontin (*SPP1*) communicated with *CD44* expressing T cells and macrophages, and these signals were identified exclusively in CLAD-BOS (Figure 5C and Supplemental Figure 9C). Osteopontin is a predictive serum biomarker for cGVHD development and has roles in fibroblast proliferation, angiogenesis, and leukocyte adhesion (72, 73).

Unexpectedly, we also observed that CLAD-BOS was enriched for potential antifibrotic intercellular communications. HGF, via binding of MET, may attenuate fibrogenesis by promoting fibroblast apoptosis and endothelial and epithelial cell proliferation and survival (74, 75). Signaling from AF1-expressing *HGF* with *MET* on epithelial and endothelial subsets was stronger in CLAD-BOS than NC, and pericyte signaling only occurred in CLAD-BOS (Supplemental Figure 9, D–F). Supporting these observations, pathways involved in wound healing, tissue and extracellular matrix remodeling, response to fibrosis-associated cytokines and growth factors, and collagen organization and metabolism were enriched in AF1 in CLAD-BOS compared with NC (Supplemental Figure 9G).

Reflecting cell-cell interaction modeling and pathway analysis, gene expression profiles in CLAD-BOS stromal cells were enriched for both pro- and antifibrotic programs. For AF1 and pericytes (Figure 5D, Supplemental Figure 9H, and Supplemental Tables 8 and 9), CLAD-BOS lungs were enriched for genes implicated in the pathogenesis of fibrotic lung disease, including those encoding collagens, matricellular proteins (*POSTN*, *TNC*, *SPARC*) (76–78), protease inhibitors (*SERPINE1*, *TIMP1*) (79, 80), hyaluronic acid synthetases (*HAS2*) (81), insulin-like growth factor binding proteins (*IGFBP7*) (82), phosphodiesterases (*PDE4D*) (83), proteoglycans (*LUM*, *VCAN*) (84, 85), and the TGF-β induced transcription factor *EGR1* (86).

FAP is a serine protease expressed primarily in fibroblasts in disease and healing wounds, but not in healthy tissues (87). *FAP* was upregulated in AF1 in CLAD-BOS, and expression was low in CD45^–^ cells irrespective of condition (Figure 5, D–F). Surprisingly, even in these samples from patients with end-stage fibrosis, these profibrotic programs were counterbalanced by genes such as *MMP19* and *PTGS2* in AF1 (Figure 5D and Supplemental Table 8) and *THY1*, *NR4A1*, and *IGFBP4* in pericytes (Supplemental Figure 9H and Supplemental Table 9), which may attenuate fibrogenesis in fibrotic lung disease (88–91). In summary, both cell-cell interactions and stromal cell gene expression profiles were enriched for potentially fibrogenic and antifibrotic programs in CLAD-BOS.

### The cGVHD-BOS transcriptome shares features with that of CLAD-BOS.

CLAD- and cGVHD-BOS share a common clinical and histopathologic phenotype and develop in the setting of allo-reactivity. Therefore, we predicted cGVHD-BOS would also be enriched for TRM and genes and pathways promoting macrophage chemotaxis and proliferation and tissue fibrosis, suggesting a shared pathogenesis with CLAD-BOS. We similarly compared samples from patients undergoing lung transplantation for cGVHD-BOS (*n* = 3) with normal age- and sex-matched controls (NC) (*n* = 3) (Figure 1A and Supplemental Table 1).

**Figure 6:**
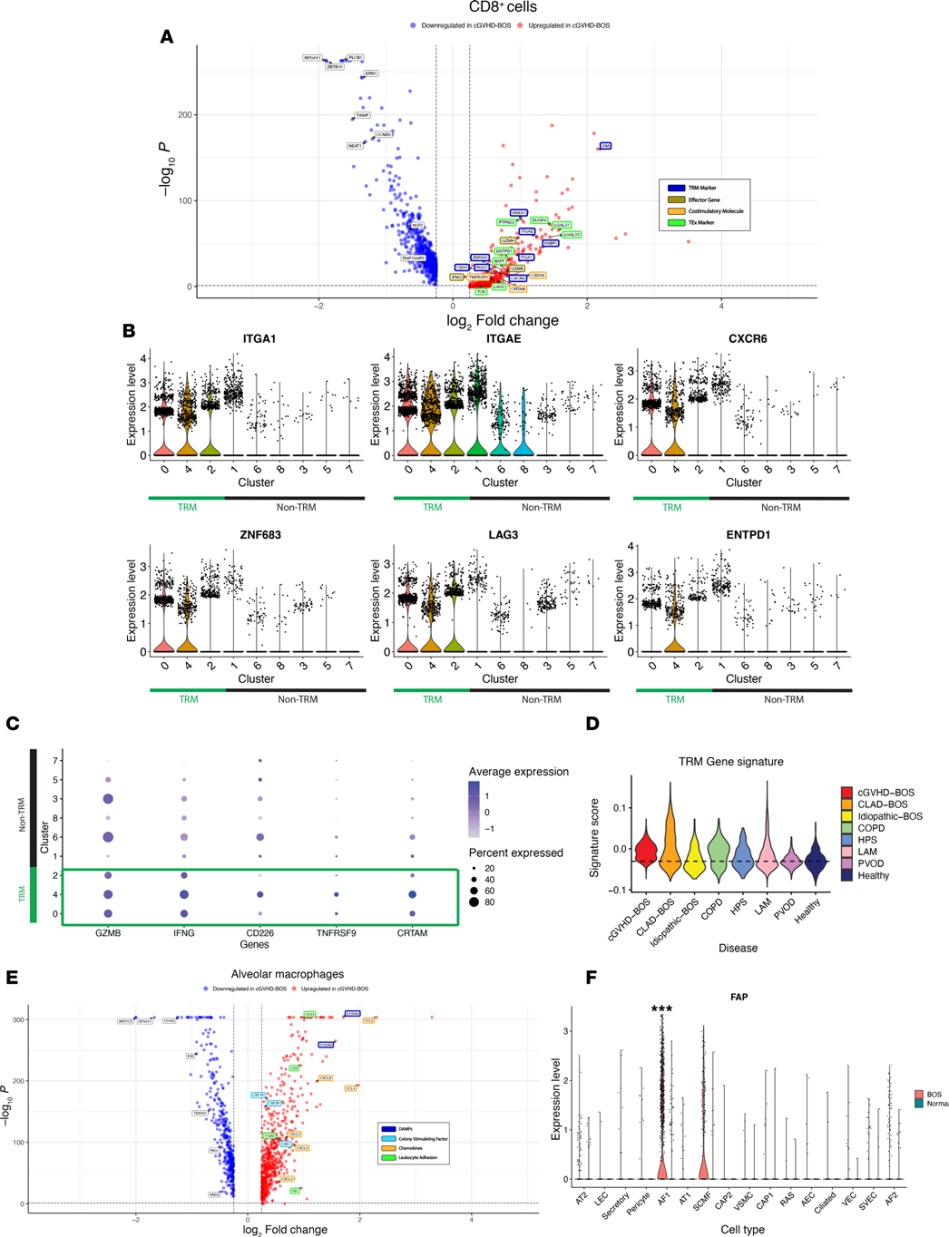
The chronic graft versus host disease BOS (cGVHD-BOS) transcriptome shares features with CLAD-BOS. (**A**) Volcano plot illustrating upregulation of genes for canonical TRM markers, effector function proteins, costimulatory molecules, inhibitory receptors, and transcription factors associated with T cell exhaustion (TEx) in CD8^+^ T cells from cGVHD-BOS compared with NC. Differences in gene expression were quantified using log_2_ fold change, and statistically significant differences were identified using the Wilcoxon rank sum test and a Bonferroni-corrected *P* < 0.05 (**B**) Violin plots for cluster-based expression of canonical TRM markers and TEx genes in CD8^+^ T cells in cGVHD-BOS with TRM clusters highlighted in green. (**C**) Dot plot with scaled expression of effector function protein and T cell costimulatory molecule genes by cluster in CD8^+^ T cells in cGVHD-BOS, with TRM clusters outlined in green. (**D**) Violin plot with TRM signature scores for CLAD- and cGVHD-BOS, NC, and 5 chronic lung diseases from the PENN-CHOP Lung Biology Institute Human Lung Tissue Bank. COPD, chronic obstructive pulmonary disease; HPS, Hermansky-Pudlak Syndrome; LAM, lymphangioleiomyomatosis; PVOD, pulmonary veno-occlusive disease. (**E**) Volcano plot illustrating differential expression of genes encoding damage-associated molecular patterns (DAMPs), colony stimulating factors, chemokines, and leukocyte adhesion proteins in alveolar macrophages in cGVHD-BOS compared with NC. (**F**) Violin plot showing *FAP* expression by CD45^–^ cell type, stratified by condition. ***P < 0.001 by Wilcoxon rank sum test, Bonferroni-corrected.

cGVHD-BOS demonstrated marked expansion of CD4^+^ and CD8^+^ T cells (Supplemental Figure 10, A and B) and, like CLAD-BOS, larger stromal and endothelial cell and smaller epithelial cell fractions compared with NC (Supplemental Figure 10, C and D). As in CLAD-BOS, CD8^+^ T cells from cGVHD-BOS were enriched for TRM markers and genes associated with TEx, effector function, and T cell activation (Figure 6A and Supplemental Table 10). TRM were expanded in cGVHD-BOS, where a larger fraction of CD8^+^ T cells expressed *ITGAE* (37%) compared with NC (16%). Mirroring CLAD-BOS, on reclustering of CD8^+^ T cells, TRM clusters (clusters 0, 4, and 2) had the highest expression of TEx (Figure 6B), effector function (Figure 6C), and T cell stimulation/activation genes (Figure 6C). Since all 3 patients with cGVHD-BOS received HLA-matched transplants, we could not employ HLA typing to resolve donor from recipient. However, 2 male patients with cGVHD-BOS underwent sex-mismatched transplants, and most lung-derived CD8^+^ T cells were of donor (female) origin in both cases (Supplemental Figure 10E).

To investigate the role of TRM in BOS compared with other lung diseases, we calculated TRM signature scores using genes identified by Kumar and colleagues as enriched in human lung TRM (Supplemental Table 11) (33) for CLAD- and cGVHD-BOS, NC, and 5 chronic lung diseases from the PENN-CHOP Lung Biology Institute Human Lung Tissue Bank. CD8^+^ T cells from CLAD- and cGVHD-BOS had higher TRM signature scores compared with other diseases in our databank and NC (Figure 6D). The signature score was also elevated in COPD, consistent with previous reports linking TRM to its pathogenesis (92, 93). Of interest, TRM scores were higher in CLAD- and cGVHD-BOS than in idiopathic BOS (Figure 6D), which occurs spontaneously in native lungs in the absence of an alloreactive immune response. This suggests that, compared with other lung disease, TRM are enriched in CLAD- and cGVHD-BOS and may be implicated in their pathogenesis.

As observed in CLAD-BOS, aMac in cGVHD-BOS had upregulation of genes encoding chemokines, adhesion molecules, and colony stimulating factors and receptors compared with NC (Figure 6E). Reflecting differential gene expression analysis, gene set enrichment analysis (GSEA) demonstrated enrichment of pathways promoting cell-cell adhesion, chemotaxis, and cytokine response in aMac (Supplemental Figure 10F). Expanding on our findings from CLAD-BOS, where aMac were primarily recipient derived, aMac in cGVHD-BOS were mostly donor derived (Supplemental Figure 10G). As in CLAD-BOS, genes implicated in fibrosis were counterbalanced by potentially antifibrotic genes in AF1 in cGVHD-BOS (Supplemental Figure 10H and Supplemental Table 12). Likewise, *FAP* expression was highest in AF1 and secondary crest myofibroblasts (SCMF) in cGVHD-BOS (Figure 6F).

### TEx, chemokine, and matricellular protein gene expression differ in CLAD- and cGVHD-BOS.

Next, we compared CLAD-BOS directly to cGVHD-BOS. aMac were expanded in CLAD-BOS, and CD8^+^ and CD4^+^ T cells were more abundant in cGVHD-BOS (Supplemental Figure 11A). For CD45^–^ cells, there were more endothelial cells in patients with CLAD-BOS and more epithelial cells in cGVHD-BOS (Supplemental Figure 11B). Although TEx genes were upregulated in TRM in both conditions compared with NC, several were more highly expressed in CLAD-BOS CD8^+^ITGAE^+^ T cells than in cGVHD-BOS (Supplemental Figure 11C and Supplemental Table 13). Except for increased expression of *GNLY* in CLAD-BOS TRM, there were no significant differences in T cell costimulatory or effector function gene expression. The aMac chemokine repertoires differed in CLAD- and cGVHD-BOS (Supplemental Figure 11D and Supplemental Table 14). Genes and pathways associated with response to TNF-α and IFN-γ signaling were upregulated in aMac in cGVHD-BOS compared with CLAD-BOS (Supplemental Figure 11, D and E). Lastly, genes for matricellular proteins (*POST*, *SPARC*) were upregulated in CLAD-BOS, and collagen encoding gene expression differed by condition (Supplemental Figure 11F and Supplemental Table 15). Taken together, direct comparison of CLAD- and cGVHD-BOS lungs revealed enrichment of TEx genes in CLAD-BOS TRM and differences in chemokine, collagen, and matricellular protein gene repertoire.

### mIF confirms a TRM phenotype with heterogenous spatial distribution in BOS lungs.

**Figure 7:**
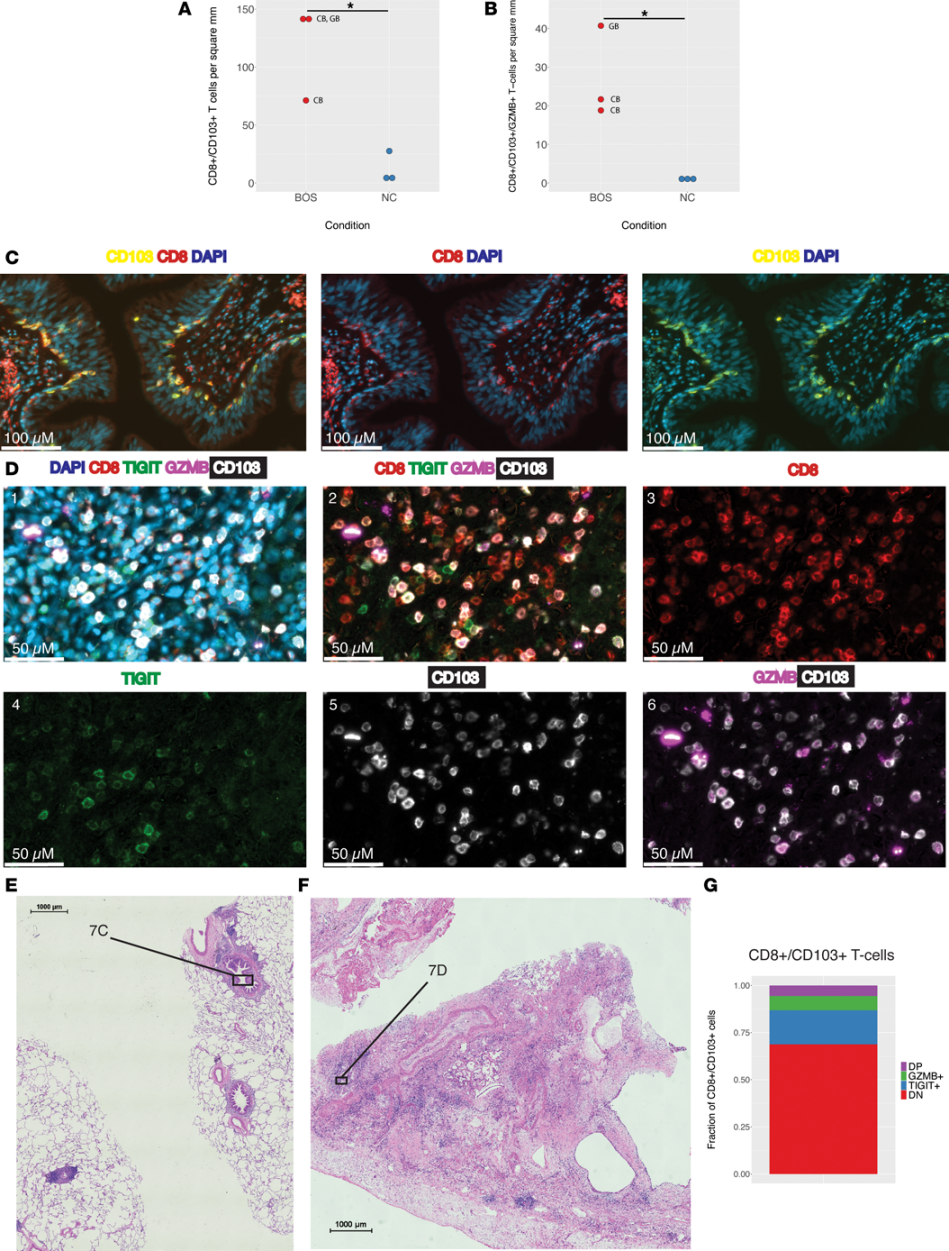
Multiplex immunofluorescence (mIF) imaging confirms expansion of TRM in BOS lungs with heterogenous spatial distribution. (**A** and **B**) Quantification of CD8^+^CD103^+^ and CD8^+^CD103^+^GZMB^+^ cells by sample for BOS (*n* = 3) versus NC (*n* = 3). Cell segmentation and marker quantification were performed on the entire 9 × 9 mm section using Visiopharm Oncotopix Discovery software. To normalize for differences in tissue quantity per section, absolute cell counts were divided by tissue area (mm^2^) calculated in QuPath. CB, CLAD-BOS; GB, cGVHD-BOS. Statistical comparison of means was performed using the student’s 1-tailed unpaired *t* test. *P < 0.05. (**C**) mIF images at 20× power illustrating infiltration of a terminal bronchiole by CD8^+^CD103^+^ T cells in a patient with CLAD-BOS. (**D**) mIF images at 20× power for a different CLAD-BOS patient with advanced fibrosis illustrating. From left to right: tissue expression of CD8, CD103, GZMB, and TIGIT with and without DAPI nuclear staining; CD8; TIGIT; CD103; and granzyme b and CD103. (**E** and **F**) H&E sections annotated with locations of mIF images from **C** and **D**, respectively. (**G**) Fraction of CD8^+^CD103^+^ cells that are negative for TIGIT and granzyme B (DN), positive for both (DP), and expressing either marker alone in the same patient with CLAD-BOS from **D**. Scale bars: 100 μM (**C**), 50 μM (**D**), 1000 μM (**E** and **F**).

We performed mIF on lung tissues for a subset of patients from the scRNA-Seq analysis with CLAD- (*n* = 2) and cGVHD-BOS (*n* = 1) compared with NC (*n* = 3). Representative H&E images from serial sections are provided for BOS lungs (Figure 7, E and F, and Supplemental Figure 12, B and D) and NC (Supplemental Figure 13, C, D, and F). Reflecting our findings from scRNA-Seq, CD8^+^CD103^+^ T cells were expanded in patients with BOS compared with NC (mean 118 cells/mm^2^ versus 12 cells/mm^2^, *P* = 0.01) (Figure 7A) as were CD8^+^CD103^+^ T cells expressing granzyme B (mean 27 cells/mm^2^ versus 1 cell/mm^2^, *P* = 0.02) (Figure 7B).

Of interest, while TRM were expanded in BOS lungs, their location varied across samples. In 1 patient with CLAD-BOS, TRM were primarily clustered in and around terminal bronchioles with fibrotic change (Figure 7, C and E, and Supplemental Figure 12, A and B). TRM were also visualized in scarred alveolar septa in the patient with cGVHD-BOS (Supplemental Figure 12, C and D) and in densely fibrotic parenchyma bearing little resemblance to normal lung tissue in a patient with CLAD-BOS (Figure 7, D and F). In contrast, there was a paucity of TRM in normal lung terminal bronchioles (Supplemental Figure 13, A–D) and alveolar septa (Supplemental Figure 13, E and F).

In addition, TRM phenotype varied across BOS samples. TIGIT was primarily expressed in TRM from the patient with CLAD-BOS with advanced fibrosis (Figure 7, D, F and G). Mirroring our transcriptional findings, this included a TRM subset coexpressing TIGIT and granzyme B (Figure 7G). These observations highlight that BOS is characterized by profound ongoing inflammation, driven in part by an infiltrate of TRM.

## Discussion

BOS after either lung or hematopoietic stem cell transplantation has a dismal prognosis with limited treatment options. An improved understanding of disease pathogenesis in human lung is necessary to identify targets for the development of new therapies. Using lung explants, we performed deep transcriptomic phenotyping of patient-derived samples to elucidate genes, pathways, and intercellular communications implicated in BOS pathogenesis. Although limited, our analysis revealed striking similarities in the cellular and molecular pathogenesis of CLAD- and cGVHD-BOS, which were notably distinct from other forms of chronic lung disease.

Despite a heavily fibrotic milieu with destruction of the normal lung architecture, we found evidence of a persistent inflammatory infiltrate in patients with end-stage CLAD- and cGVHD-BOS. This included a TRM population that was overrepresented in both CLAD- and cGVHD-BOS with a unique transcriptomic signature compared with other lung diseases across a large database and NC.

Using mIF microscopy, we validated these findings at the protein level, while highlighting heterogeneity of TRM phenotype and tissue localization across BOS samples. Previous studies describe localization of TRM to small airways in healthy lung grafts after transplant (94). Similarly, TRM were distributed along fibrotic alveoli and bronchi in CLAD- and cGVHD-BOS. However, TRM were also highly concentrated in densely fibrotic regions lacking normal airway structures.

In health, TRM persist following cognate antigen recognition to provide the first line of defense against microbes and malignancy (95, 96). However, TRM can be deleterious and are implicated in the pathogenesis of autoimmune disease (97, 98), transplant rejection (94, 99), and acute GVHD (44). Our data in CLAD-BOS build on previous findings implicating cytotoxic TRM in rejection of solid organ transplant (45, 94, 99–101). In human intestinal and kidney grafts with chronic rejection, TRM expressed TNF-α, IFN-γ, granzyme B, and IL-2 and were potently cytotoxic on ex vivo stimulation (99, 102), as were TRM from a murine model of renal graft rejection despite high PD-1 expression (45). Although our study lacks functional validation, it is possible that TRM in BOS, as in other forms of chronic rejection, are persistently cytotoxic to host tissues despite chronic stimulation by alloantigen and consequent IR expression.

Highlighting the role of alloreactive recipient–derived TRM in pathogenesis, the risk of chronic rejection is higher in intestinal (102), lung (94), and renal grafts (99) with higher recipient-derived TRM. With some exceptions, TRM become primarily recipient derived by 12 months following lung transplant, and the recipient TRM fraction increases with time (94). Consistent with these observations, TRM were recipient derived in our CLAD-BOS data, where the median time between first and second lung transplant was 65 months (Supplemental Table 1). We expand these findings to cGVHD-BOS, where most TRM were derived from the hematopoietic stem cell donor. It is possible that expansion of bone marrow–derived, pathogenic TRM in BOS lung grafts is driven by response to allo-antigen. However, a limitation of our study is the absence of lung tissue from posttransplant recipients who did not have CLAD- or cGVHD-BOS.

The identification of pathogenic TRM in CLAD- and cGVHD-BOS could have therapeutic implications, since TRM may be resistant to standard immunosuppressive agents. In a mouse model of chronic rejection, cyclosporine failed to prevent the formation of TRM in kidney grafts (45), and TRM persist in bronchoalveolar lavage fluid of patients with acute rejection despite treatment with high-dose corticosteroids (103). In clinical practice, standard immunosuppression is ineffective in patients with advanced BOS (4), and based on our results, we speculate that this could be due to the inherent resistance of TRM to these therapies. This highlights the need for TRM-specific/targeted therapies for solid organ transplant rejection and pulmonary cGVHD.

In keeping with findings from CLAD-BOS and murine models of cGVHD, our data support the role of innate immunity in disease pathogenesis (1, 19). Both CLAD- and cGVHD-BOS lungs were enriched for genes associated with macrophage chemotaxis, adhesion, and proliferation. Direct comparison of CLAD- and cGVHD-BOS pathways showed enrichment of IFN-γ and TNF-α signaling in aMac in cGVHD-BOS. Of note, the TNF-α inhibitor etanercept has efficacy in steroid refractory cGVHD based on small, single-center studies (104, 105). Consistent with previous studies (106–108), aMac in CLAD-BOS were primarily recipient derived, and we expand these findings to cGVHD-BOS, where aMac were mostly donor derived. Taken together, in both CLAD- and cGVHD-BOS, aMac are replaced over time by bone marrow–derived macrophages.

Of interest, both immune and nonimmune cell subsets contributed to a chemokine-rich inflammatory milieu. We expand on similar findings in COPD by identifying AT2 and RAS cells as the principal sources of CXCL8 and CXCL1 secretion in CLAD-BOS (62). CSF1/CSF1R signaling is critical to macrophage proliferation and fibrogenesis in murine models of cGVHD-BOS (19), and in humans, the CSF1R inhibitor axatilimab recently received regulatory approval for steroid refractory cGVHD (109). Our data confirm enrichment of this pathway in humans with both CLAD- and cGVHD-BOS and provide preliminary rationale for extending this therapeutic intervention to CLAD-BOS.

Cell-cell/ligand-receptor interaction modeling predicted robust crosstalk between stromal cells and dysregulated macrophage and T cell subsets in CLAD- and cGVHD-BOS. Interestingly, these immune-stromal intercellular communications were characterized by both pro- and antifibrotic programs. These findings raise the future prospect to intervene therapeutically in the dynamic balance of pro- and antifibrotic forces in CLAD- and cGVHD-BOS.

FAP is a serine protease that is expressed in fibroblasts and is associated with pathophysiologic tissue remodeling, including in inflammatory and fibrotic diseases and tumor stroma (87, 110). Consistent with this precedent, we demonstrate that *FAP* expression is enriched in CLAD- and cGVHD-BOS stromal cells. Leveraging this specificity, preclinical investigations have identified FAP as a target for chimeric antigen receptor (CAR) T cell therapy and antigen-directed drug delivery in malignant and fibrotic disease (87, 111, 112). Our findings may provide an initial rationale for investigating the role of FAP-targeted therapies in CLAD- and cGVHD-BOS.

In this study, we describe the breadth of immune, epithelial, and stromal dysfunction in lung explants from 4 patients with end-stage CLAD-BOS and 3 with cGVHD-BOS. While acknowledging the limitations of our primarily descriptive approach to pathogenesis, we present this work as a hypothesis-generating study for future deeper analysis and validation. We are particularly intrigued by (a) the potential role of TRM in BOS pathogenesis and rationale for TRM specific therapies; (b) preliminary rationale for CSF1R inhibition in CLAD-BOS; (c) a possible compensatory antifibrotic response in BOS, providing preliminary justification for therapies shifting the balance from a pro- to antifibrotic milieu; and (d) similar transcriptomes in CLAD- and cGVHD-BOS, suggesting a shared pathogenesis and, potentially, common therapeutic targets. Overall, this study provides insight into the pathogenesis of this devastating disease and opens multiple avenues of investigation for improving the standard of care for patients with BOS.

## Methods

### Sex as a biological variable.

All patients with BOS were male, since there were no lung explants available from female patients in this small dataset. Sex was not considered as a biological variable.

### Tissue preparation and scRNA-Seq.

Lungs were surgically removed at the time of bilateral orthotopic lung transplant among patients who had previously undergone lung transplant or ASCT and whose primary indication for transplant was BOS, confirmed radiographically and physiologically. NC lung was derived from unused human lung donors and was procured at the time of organ donation and confirmed by pathological review (113, 114). Samples were prepared and CD45^+^ and CD45^–^ cells were isolated as previously described (113, 114). The 10X Genomics Chromium Controller platform with Chromium Next GEM Single Cell 3′ Reagent Kits v3.1 was used to assemble 3′ gene expression libraries. Sequencing was performed on an Illumina Novaseq 6000 instrument.

### scRNA-Seq data analysis.

Transcripts were mapped to the GRCh38 human reference genome, and feature-barcode matrices were generated using Cell Ranger count v7.0.1 from 10X Genomics. All further downstream data analysis was performed in R v4.2.1 unless otherwise indicated. The SoupX package v1.6.2 was used to estimate and remove contaminating ambient RNA (115). Contamination fractions were approximated for each sample by estimating the expression of complement genes C1QA and C1QB in nonexpressing cells. Doublets were removed using the DoubletFinder package v2.0.3 (116).

Quality control filtering, normalization, data integration and batch correction, principal component analysis, clustering, and uniform manifold approximation and projection (UMAP) were performed separately for the CD45^+^ and CD45^–^ cells using the Seurat package v4.3. Quality filtering parameters included > 500 transcripts per cell, > 250 genes per cell, log_10_ genes per unique molecular identifier > 0.80, mitochondrial percentage < 20%, and genes expressed in > 10 cells.

Normalization was performed using SC transform for UMAP visualizations (117); log transformed counts were used for differential gene expression, pathway, and cell-cell/ligand receptor interaction analyses. SC transform normalized samples were integrated using canonical correlation analysis. The clustree package was used to identify optimal cluster resolutions for UMAP visualizations (118). Reference-based cell type annotation of CD45^+^ and CD45^–^ cells was performed with Azimuth, using the Human Lung Cell Atlas and LungMAP Cell Cards reference data sets, respectively (26, 27, 119). The following cell types were excluded from downstream analysis due to small sample size: CCL3^+^ aMac, migratory DCs, plasma cells, plasmacytoid DCs, airway smooth muscle cells, goblet cells, ionocytes, mesothelial cells, pulmonary neuroendocrine cells, submucosal gland cells, tuft cells, proliferating T cells, and proliferating macrophages.

Volcano plots and feature plots illustrating differential expression were designed using the EnhancedVolcano v1.14.0 (120) and scCustomize v1.1.1 packages (121), respectively. UMAP coexpression density plots were created using the Nebulosa v1.6.0 package (122). Cell sex and HLA type were determined using the speckle package v0.0.3 (123) and command line–based arcasHLA v0.6.0 package, respectively (124). Clinically obtained, ground truth HLA types using serologic nomenclature were compared with predicted HLA types in molecular nomenclature generated by arcasHLA for CD8^+^ T cells, aMac, and AT2. For donor lungs, only HLA types for A, B, and DR alleles were available. In 1 patient, all CD45^+^ cells were typed, and for a second patient all CD45^–^ cells were typed, as there were insufficient alignments for full HLA typing in CD8^+^ T cells and AT2 alone, respectively.

The TRM gene signature from Kumar and colleagues (33) included 226 genes upregulated in human lung TRM compared with non-TRM with log_2_ fold change ≥ 1 and FDR ≤ 0.05. Scores were calculated using the AddModuleScore function in Seurat for CLAD- and cGVHD-BOS, NC, and 5 chronic lung diseases from the PENN-CHOP Lung Biology Institute Human Lung Tissue Bank. GSEA was performed in the clusterProfiler package v4.7.1 using the molecular signatures database (MSigDB) C5 gene ontology gene sets (125, 126).

Cell-cell/ligand-receptor interactions between cells were evaluated with the CellChat package v1.6.1 (127). All cell populations were downsampled to 300 cells. Interaction strength was calculated as a function of ligand-receptor pair expression while accounting for expression of costimulatory and coinhibitory receptors and extracellular agonists and antagonists. Outgoing signal was defined as expression of the ligand for a given ligand-receptor pair, and incoming signal was defined as expression of the corresponding receptor.

### mIF processing and analysis.

Tissue was cut from the distal lung as described above, washed 2–5 times in PBS, and placed in 4% paraformaldehyde overnight. Tissue was washed in PBS, dehydrated to 100% alcohol over 12–24 hours, and embedded in paraffin, and blocks were sectioned to 9 × 9 mm and 6 μm thickness for mIF slides. All mIF was performed using the Lunaphore COMET platform. The COMET system was prepared per manufacturer guidelines, and slides were warmed at 60°C for 1 hour followed by deparaffinization with CitriSolv (Decon Labs) (3 washes × 10 minutes), 100% ethanol (2 washes × 3 minutes), 95% ethanol (2 washes × 3 minutes), and milliQ water (2 washes × 3 minutes). Antigen retrieval was performed in a pressure cooker device for 15 minutes using pH 9.5 Borg Decloaker RTU (Biocare Medical) and 3% hydrogen peroxide incubation. Blocking solution consisting of 10% donkey serum, MSB, and 0.5% Tween20 (Thermo Fisher Scientific) was administered for 1 hour at room temperature. A maximum of 4 slides was loaded on the COMET platform for each run.

Staining was done per manufacturer protocol, and nucleic acid stain Hoechst 33342 trihydrochloride trihydrate (10 mg/mL, Invitrogen) was used. Elution and staining were done at 37°C. Imaging was performed using TRITC and Cy5 channels, with DAPI visualized at an exposure time of 80 ms. Detailed information on the antibodies employed and COMET platform parameters are provided in Supplemental Table 16.

The Lunaphore COMET platform acquires mIF images at a native 20× optical magnification and pixel size of 0.28 mm for a maximum slide dimension of 9 × 9 mm. TRITC and Cy5 background subtraction were performed on all mIF images in COMET Viewer software. Quantitative analysis of marker expression was performed in Visiopharm Oncotopix Discovery software v2024.07.1. To identify and quantify cell types of interest, entire 9 × 9 mm mIF slides were analyzed using the Cell Detection, AI application, which employs a proprietary deep learning method to segment and quantify cells. The Phenoplex Guided Workflow was employed to establish manual thresholds for marker positivity, aided by quality control instruments like scatter plots to inspect marker coexpression at single cell resolution and to ultimately quantify marker expression at the single cell level. Tissue area (mm^2^) for the entire 9 × 9 mm slide was calculated using QuPath (128). To normalize between samples in tissue quantity per slide, total cell number was divided by tissue area. Quantitative data were exported for further analysis in R v4.2.1, and dot plots were generated using the ggplot2 package v3.4.1.

### Statistics.

Differences in gene expression between conditions were quantified using log_2_ fold change, and statistical significance calculated with the Seurat FindMarkers function using the Wilcoxon Rank Sum Test. Statistical significance for DEG was defined as a Bonferroni-corrected *P* < 0.05. Statistically significant interactions between cell groups were identified using a 1-sided permutation test with significance threshold of *P* < 0.05. For GSEA in clusterProfiler, *P* values for normalized enrichment scores were calculated using permutation testing as previously described (129) and corrected for multiple comparisons using the Benjamini-Hochberg method. Differences in mean cell quantity/mm^2^ between conditions were assessed using the Student’s 1-tailed unpaired *t* test, with a significance threshold of *P* < 0.05.

### Study approval.

NC used in this study were from deidentified, nonused lungs donated for organ transplantation through the Prospective Registry of Outcomes in Patients Electing Lung Transplantation (PROPEL), approved by the Penn IRB. Informed consent was provided by next of kin or health care proxy in accordance with NIH and institutional procedures. BOS tissue was obtained from participants enrolled in PROPEL at Penn. This study was approved by the Penn IRB, and written informed consent was obtained from all patients with BOS prior to enrollment. All patient-identifying information was removed prior to use. While this does not meet current NIH definition for human research, the reported experiments followed all guidelines, regulations, and institutional procedures required for human research.

### Data availability.

Raw and filtered count data for 10X Genomics single-cell experiments are deposited in the Gene Expression Omnibus (GEO) (accession no. GSE290834) and FASTQ files in the LungMap database of Genotypes and Phenotypes (dbGaP). Values for all main text and Supplemental figures depicting quantitative data can be found in the accompanying Supporting Data Values file.

## Author contributions

PWM, MCB, and SG designed the study and analyzed data. PWM performed all bioinformatic analysis. ANL performed tissue preparation and scRNA-Seq of BOS and NC samples and manual mIF antibody testing. BCR performed manual mIF antibody testing and all COMET experiments. MS and JDP provided bioinformatic support. ARP, YY, SZ, all performed histological sample preparation, H&E imaging, and processing for mIF sample imaging. JDC, JMD, and EC all contributed to patient enrollment, study design, and tissue acquisition. All authors reviewed and edited the final manuscript.

## Supplementary Material

Supplemental data

Supplemental tables 1-16

Supporting data values

## Version 1 04/15/2025

In-Press Preview

## Version 2 05/22/2025

Electronic publication
