# Supplementary material for: Shared roles of immune and stromal cells in the pathogenesis of human bronchiolitis obliterans syndrome
Source: JCI Insight. 2025 Apr 15;10(10):e176596. doi: 10.1172/jci.insight.176596 (PMC12128974; doi:10.1172/jci.insight.176596)
Supplement: Supplemental data [file jciinsight-10-176596-s244.pdf]

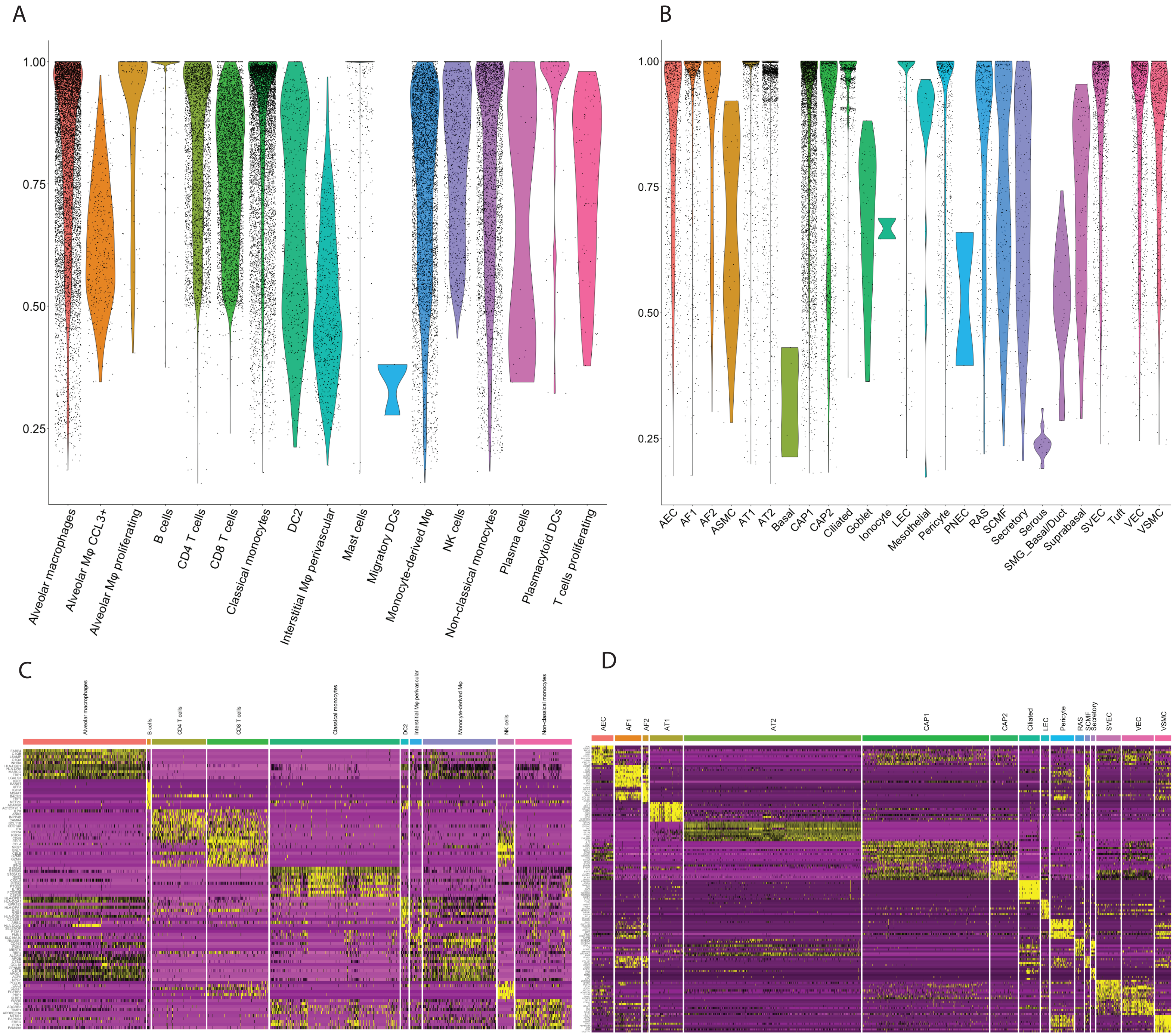

Supplementary Figure S1. Azimuth prediction scores for **(A)** CD45+ve cell types annotated with the Human Lung Cell Atlas and **(B)** CD45-ve cell types annotated with LungMAP Cell Cards. Heatmaps illustrating top 10 marker genes for **(C)** CD45+ve and **(D)** CD45-ve cell types with > 300 cells

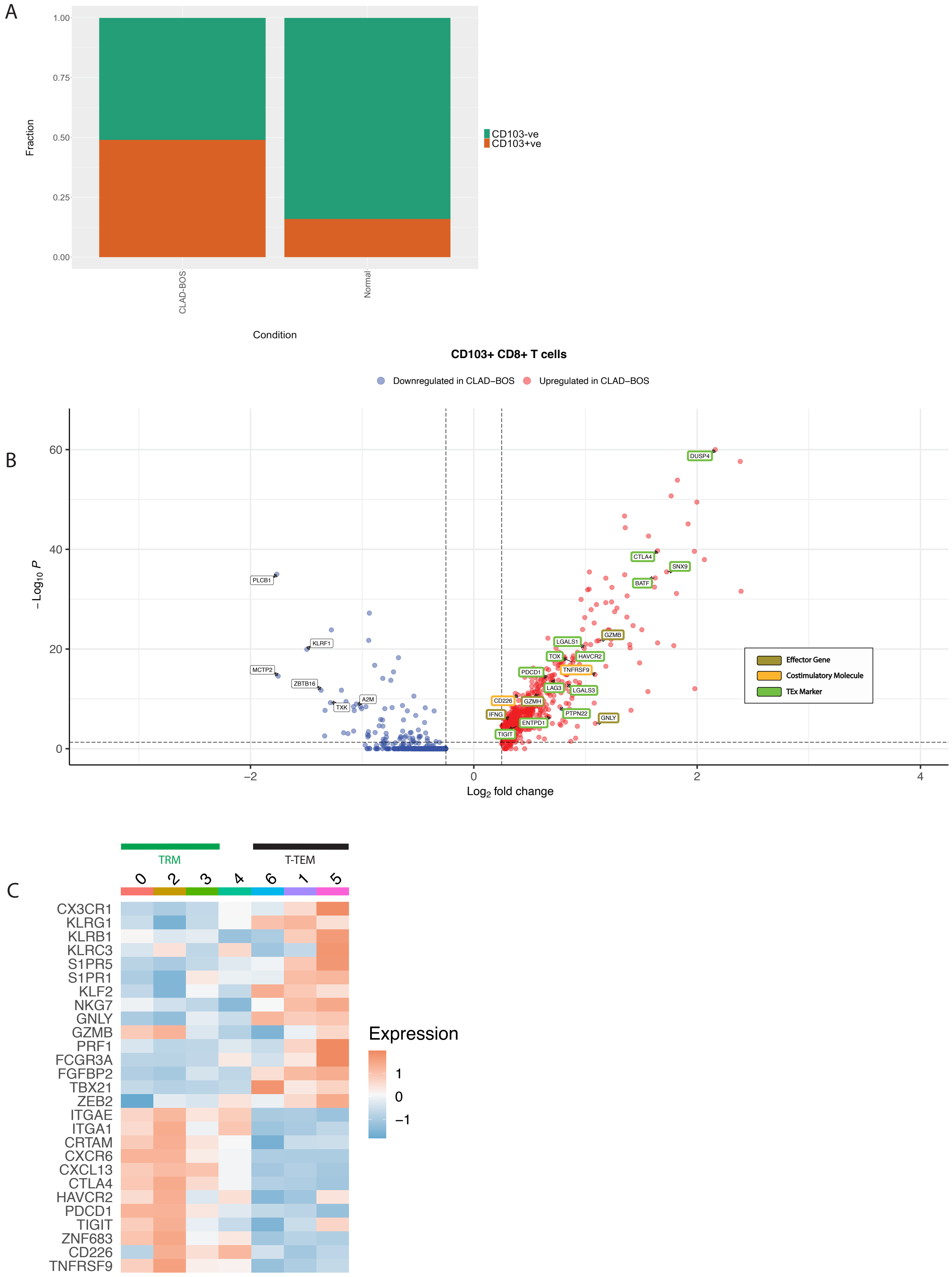

Supplementary Figure S2. (A) Bar plot illustrating fraction of CD103+ve and CD103-ve CD8+ T cells in CLAD-BOS versus normal controls (NC) **(B)** Volcano plot illustrating expression of T-cell exhaustion-associated (TEx), effector function, and T-cell activation/stimulation genes in CD103+ CD8+ T-cells in CLAD-BOS versus NC. Differences in gene expression were quantified using log2fold change and statistically significant differences were identified using the Wilcoxon Rank Sum Test and a Bonferroni corrected P-value <0.05 **(C)** Heatmap with scaled expression of genes associated with tissue resident memory T-cells (TRM) and terminally differentiated effector memory T-cells (T-TEM) in CLAD-BOS by CD8+ T-cell cluster. TRM (0, 2, and 3) and T-TEM clusters (6, 1, and 5) are highlighted with green and black bars, respectively, while cluster 4 has both TRM and T-TEM features.

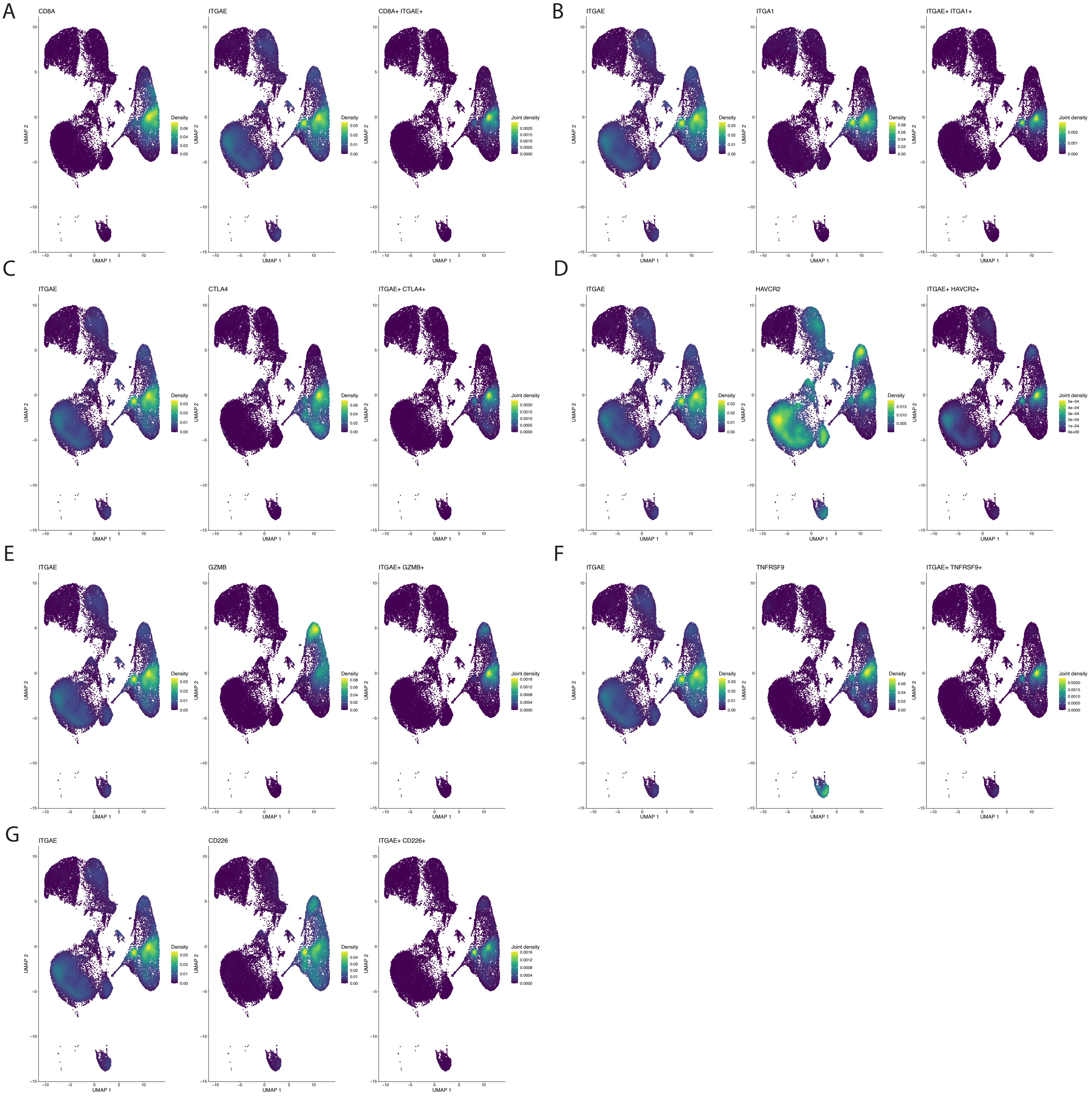

Supplementary Figure S3. UMAP density plots for CD45+ve cells in CLAD-BOS illustrating co-expression of **(A)** CD8A and ITGAE, ITGAE and **(B)** ITGA1 **(C)** CTLA4 **(D)** HAVCR2 **(E)** GZMB **(F)** TNFRSF9 **(G)** and CD226

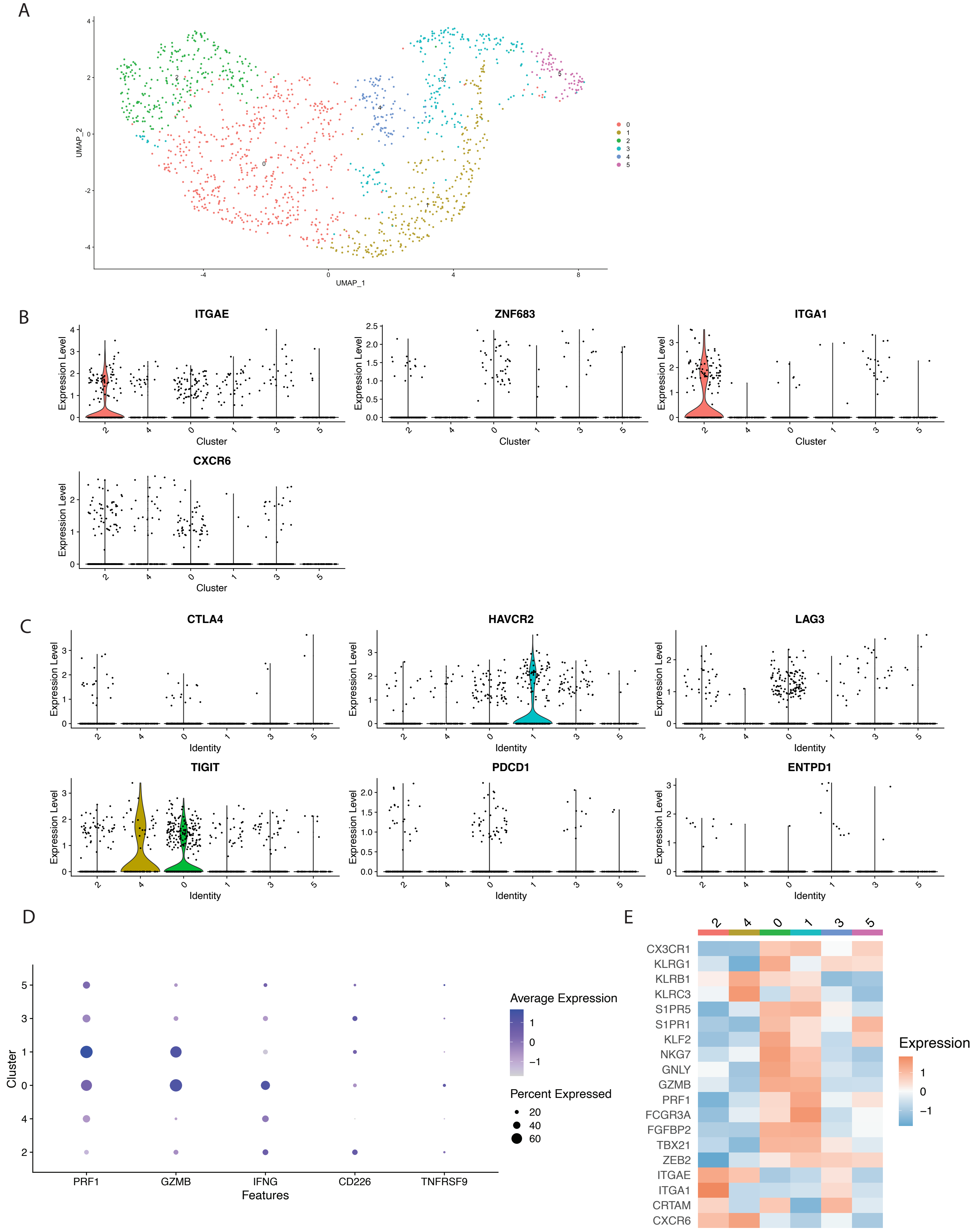

Supplementary Figure S4. (A) UMAP projection for re-clustered CD8+ T-cells from normal controls (NC) **(B)** Violin plot with expression of canonical tissue resident memory (TRM) and **(C)** inhibitory receptor genes by cluster in CD8+ T-cells from NC **(D)** Dot plot with scaled expression of effector function and T-cell stimulatory/activation genes by cluster in CD8+ T-cells from NC **(E)** Heatmap with scaled expression of genes associated with TRM and terminally differentiated effector memory T-cells in NC by CD8+ T-cell cluster

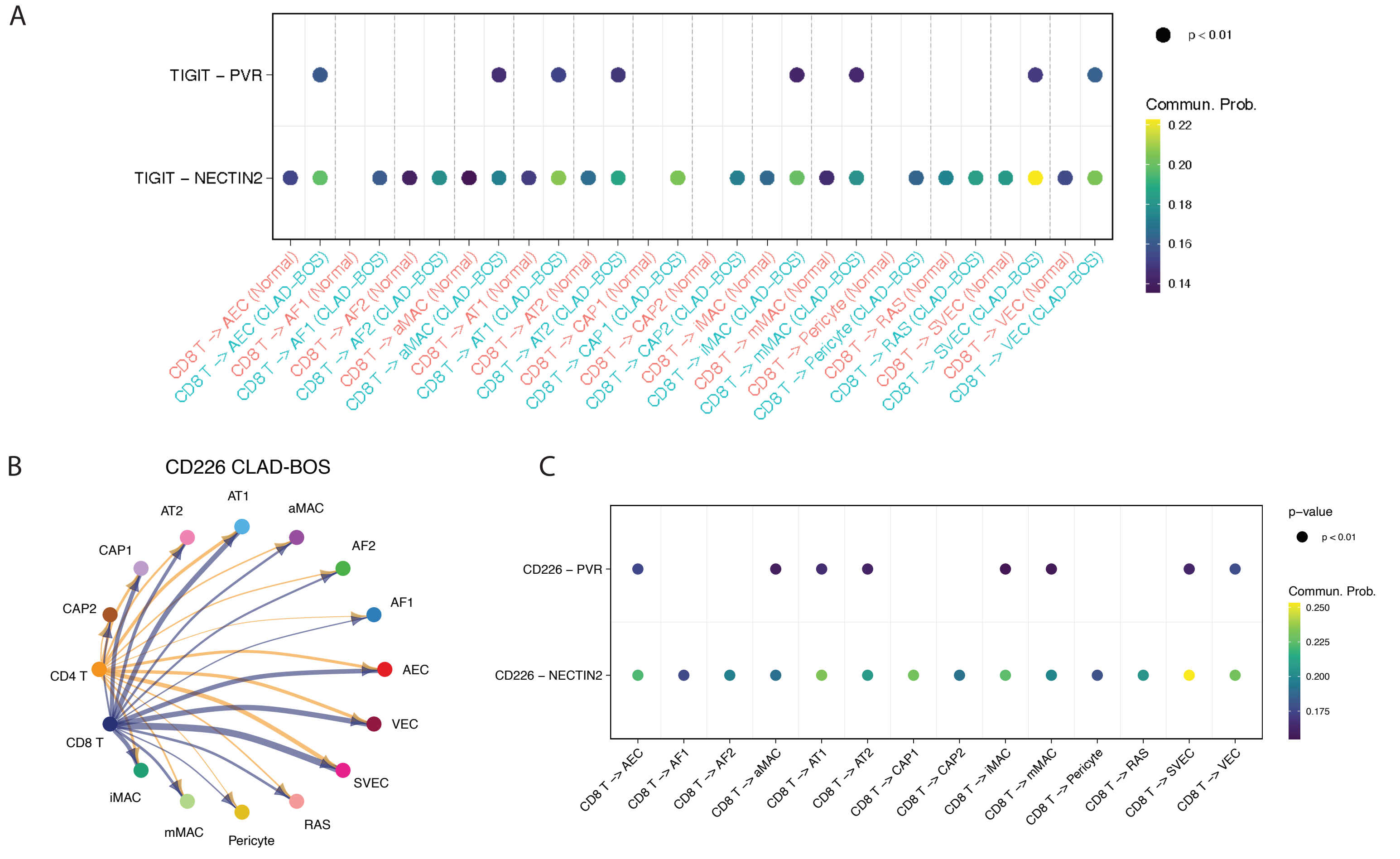

Supplementary Figure S5. (A) Dot plot illustrating ligand/receptor pairs and interaction strength for putative TIGIT signaling from CD8+ T-cells in CLAD-BOS versus normal controls (NC). Colors correspond to communication strength. P values for interactions are calculated using a one-sided permutation test **(B)** Circle plot illustrating putative CD226 signaling in CLAD-BOS lungs from CD4+ and CD8+ T-cells. This signaling did not occur in NC. Lines connect interacting cells (dots), and cells sending outgoing signal have the same color as the corresponding line. All interactions depicted are statistically significant using a one-sided permutation test with significance threshold of P-value <0.05, and thicker lines indicate stronger interactions **(C)** Dot plot illustrating ligand/receptor pairs and interaction strength in CLAD-BOS for CD8+ T-cell CD226 signaling.

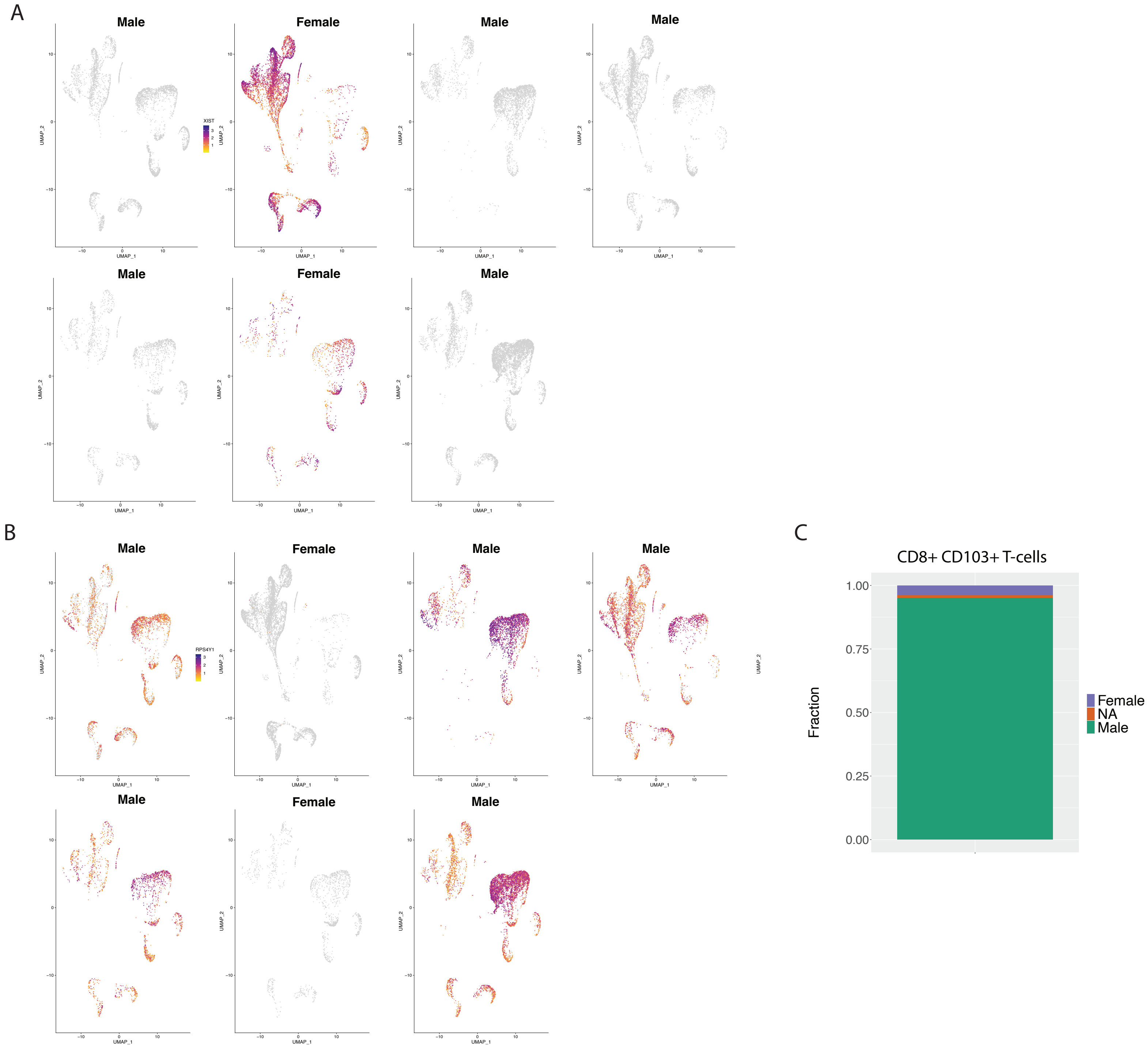

Supplementary Figure S6. **(A)** XIST and **(B)** RPS4Y1 expression in CD45-ve cells in (top row) 4 CLAD-BOS donor lungs and (bottom row) 3 normal controls **(C)** Sex of lung-derived CD8+ CD103+ T-cells in a male CLAD-BOS patient with a female lung donor using the speckle package, which employs logistic regression and multiple X and Y-associated genes to predict cell sex. NA signifies that cell sex could not be determined due to lack of expression of X and Y associated genes.

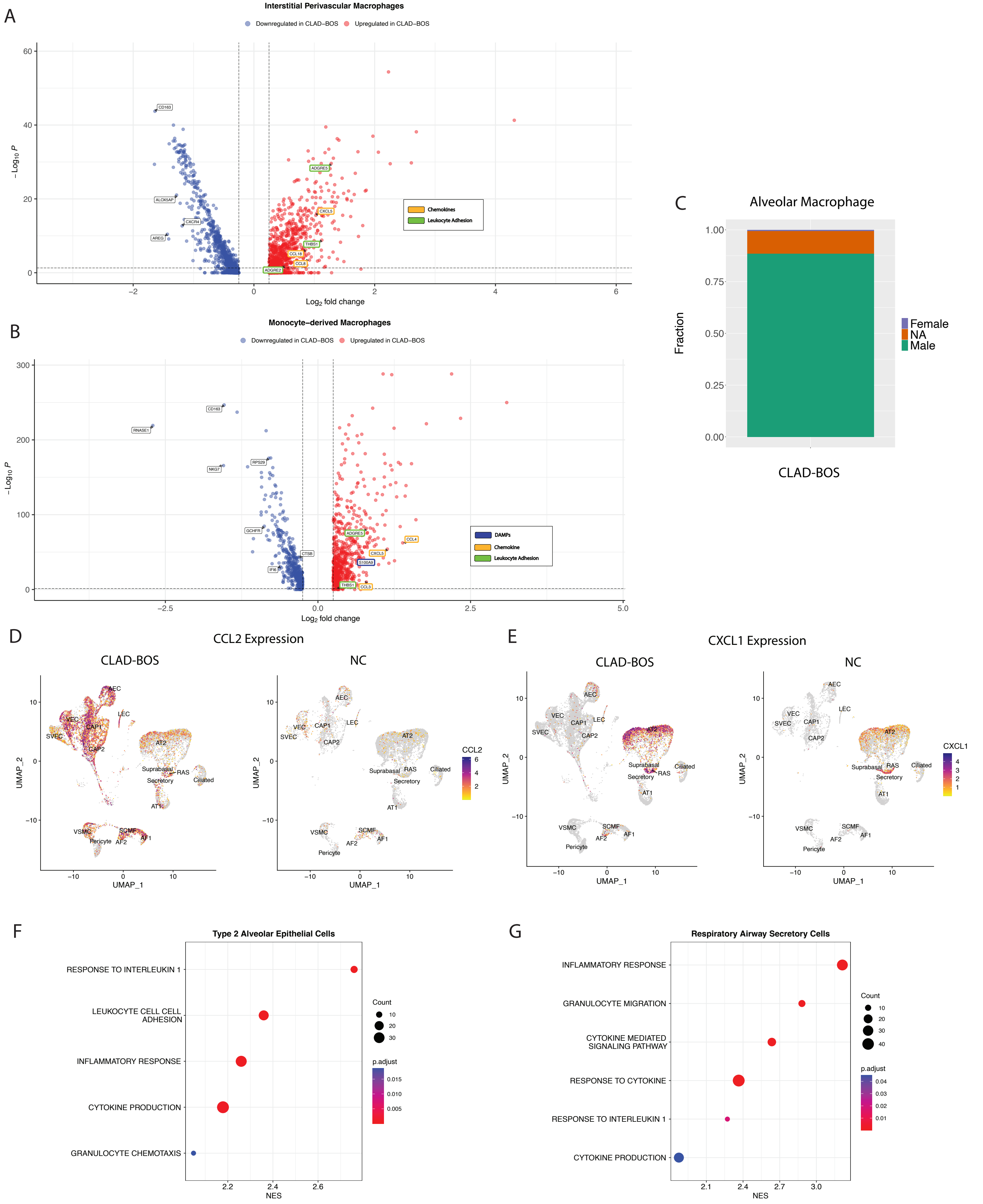

Supplementary Figure S7. (A) Volcano plot illustrating differentially expressed genes promoting macrophage chemotaxis and adhesion in interstitial perivascular macrophages and **(B)** monocyte-derived macrophages in CLAD-BOS compared to normal controls (NC). Differences in gene expression were quantified using log2fold change and statistically significant differences were identified using the Wilcoxon Rank Sum Test and a Bonferroni corrected P-value <0.05. DAMPs, damage associated molecular patterns. **(C)** Sex of lung-derived alveolar macrophages in a male CLAD-BOS patient with a female lung donor using the speckle package, which employs logistic regression and multiple X and Y-associated genes to predict cell sex. NA signifies that cell sex could not be determined due to lack of expression of X and Y associated genes. **(D)** Feature plot illustrating CCL2 and **(E)** CXCL1 expression in CD45-ve cell populations in CLAD-BOS compared to NC **(F)** Dot plot with enrichment of gene ontology (GO) terms related to chemotaxis and cytokine response in type 2 alveolar epithelial cells and **(G)** respiratory airway secretory cells in CLAD-BOS compared to NC. P-values for normalized enrichment score (NES) were adjusted for multiple comparisons using the Benjamini-Hochberg method.

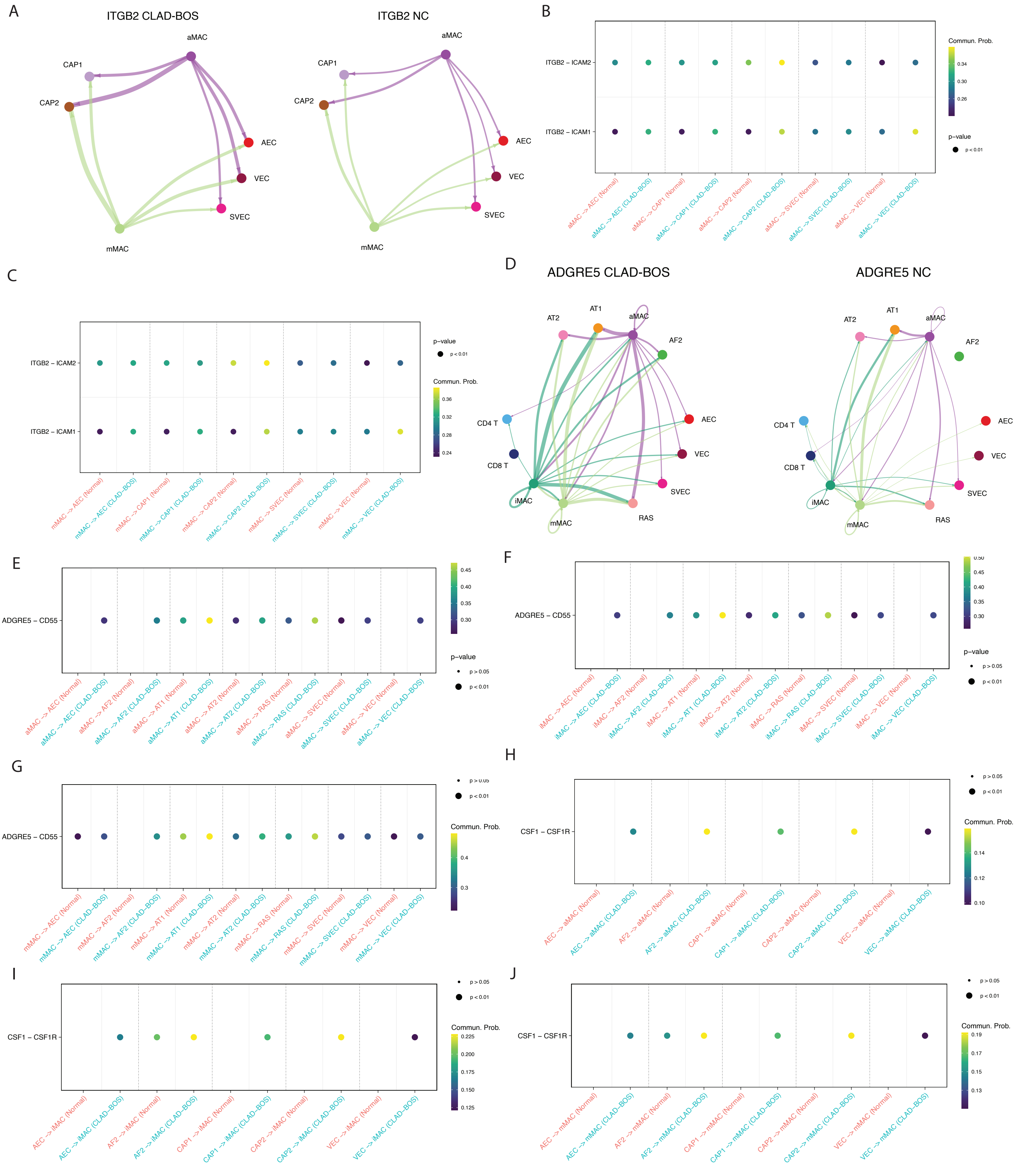

Supplementary Figure S8. (A) Circle plot illustrating interaction strength for ITGB2/LFA-1 signaling in CLAD-BOS (left) and normal control (NC) (right) lungs from aMac and mMac to endothelial cell subsets. Lines connect interacting cells (dots), and cells providing the outgoing signal have the same color as the corresponding line. All interactions depicted are statistically significant using a 1-sided permutation test with significance threshold of P-value <0.05, and thicker lines indicate stronger interactions. Dot plots illustrating ligand/receptor pairs and interaction strength in CLAD-BOS versus NC for **(B)** aMac and **(C)** mMac expressing ITGB2/LFA-1 signaling to endothelial cell subsets expressing ICAM1 and ICAM2. Colors correspond to communication strength. P values for interactions are calculated using a 1-sided permutation test **(D)** Circle plot illustrating interaction strength for ADGRE5/CD97 signaling in CLAD-BOS (left) and NC (right) lungs from aMac, iMac, and mMac to epithelial, stromal, and endothelial cell populations expressing CD55. Dot plots illustratIng ligand/receptor pairs and interaction strength in CLAD-BOS versus NC for **(E)** aMac, **(F)** iMac, and **(G)** mMac expressing ADGRE5/CD90 signaling to endothelial, stromal and epithelial subsets expressing CD55. Dot plots illustrating ligand/receptor pairs and interaction strength in CLAD-BOS for endothelial cell and AF2 CSF-1 signaling to **(H)** aMac, **(I)** iMac, and **(J)** mMac expressing CSF1R

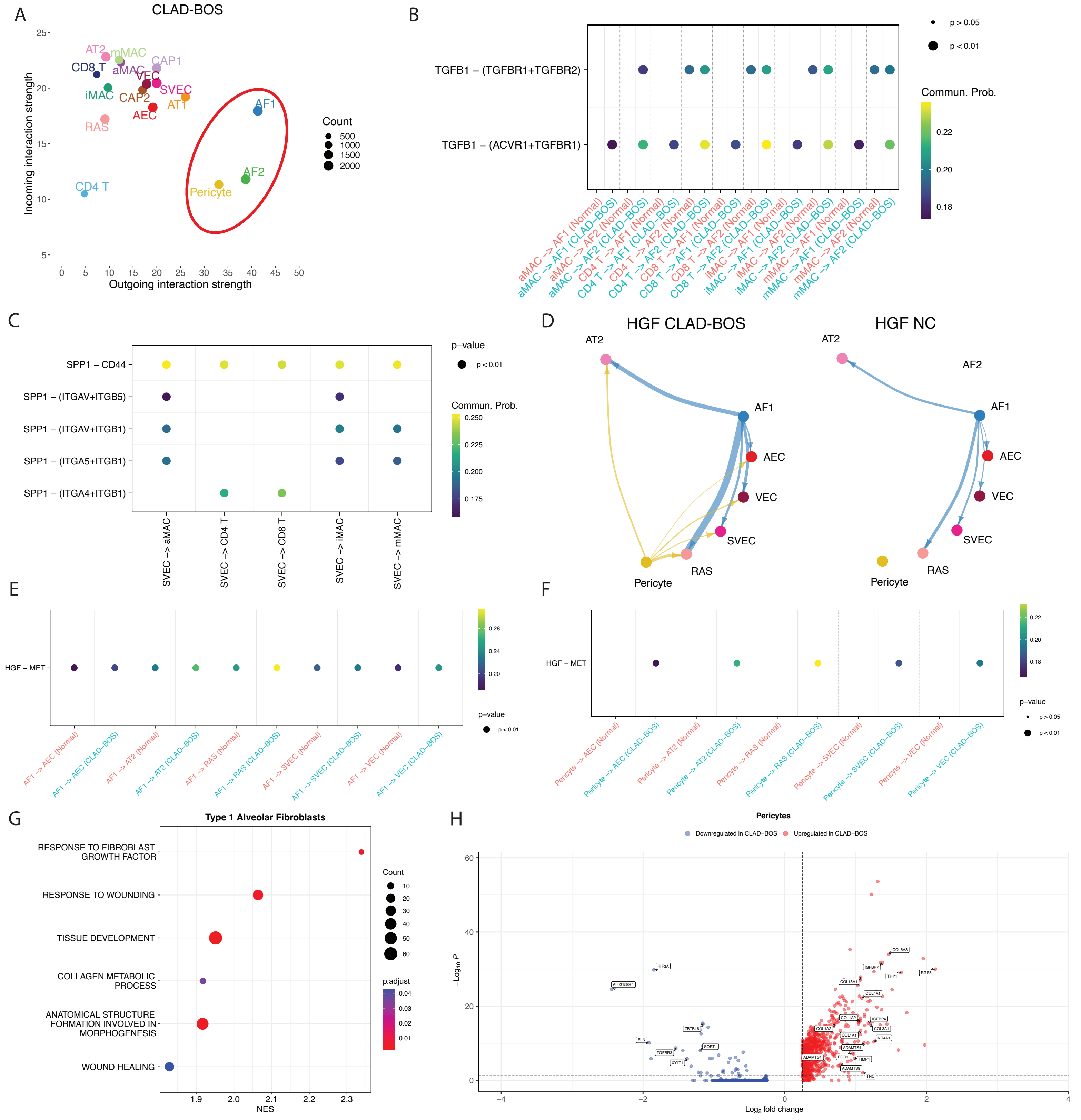

Supplementary Figure S9. (A) Incoming and outgoing interaction strengths for all cell types in CLAD-BOS. Stromal cells including pericytes, AF2, and AF1 are outlined in red **(B)** Dot plot illustrating ligand/receptor pairs and interaction strength in CLAD-BOS versus normal controls (NC) for TGF-beta signaling from macrophages and T-cells to AF1 and AF2. Colors correspond to communication strength. P values for interactions are calculated using a one-sided permutation test **(C)** Dot plot illustrating ligand/receptor pairs and interaction strength in CLAD-BOS versus NC for SPP1 signaling from SVEC to macrophages and T-cells **(D)** Circle plot illustrating interaction strength for HGF signaling in CLAD-BOS (left) and NC (right) lungs from AF1 and pericytes to endothelial and epithelial cell subsets. Lines connect interacting cells (dots), and cells providing the outgoing signal have the same color as the corresponding line. All interactions depicted are statistically significant using a 1-sided permutation test with significance threshold of P-value <0.05, and thicker lines indicate stronger interactions. Dot plots illustrating ligand/receptor pairs and interaction strength in CLAD-BOS versus NC HGF signaling from **(E)** AF1 and **(F)** pericytes to MET expressing endothelial and epithelial cells. **(G)** Dot plot with enrichment of gene ontology (GO) terms related to fibrosis and wound healing in AF1 in CLAD-BOS compared to NC. P-values for NES were corrected for multiple comparisons using the Benjamini-Hochberg method. NES, normalized enrichment score **(H)** Volcano plot illustrating expression of pro and anti-fibrotic genes in pericytes in CLAD-BOS versus NC. Differences in gene expression were quantified using log2fold change and statistically significant differences were identified using Wilcoxon Rank Sum Test and a Bonferroni corrected P-value <0.05

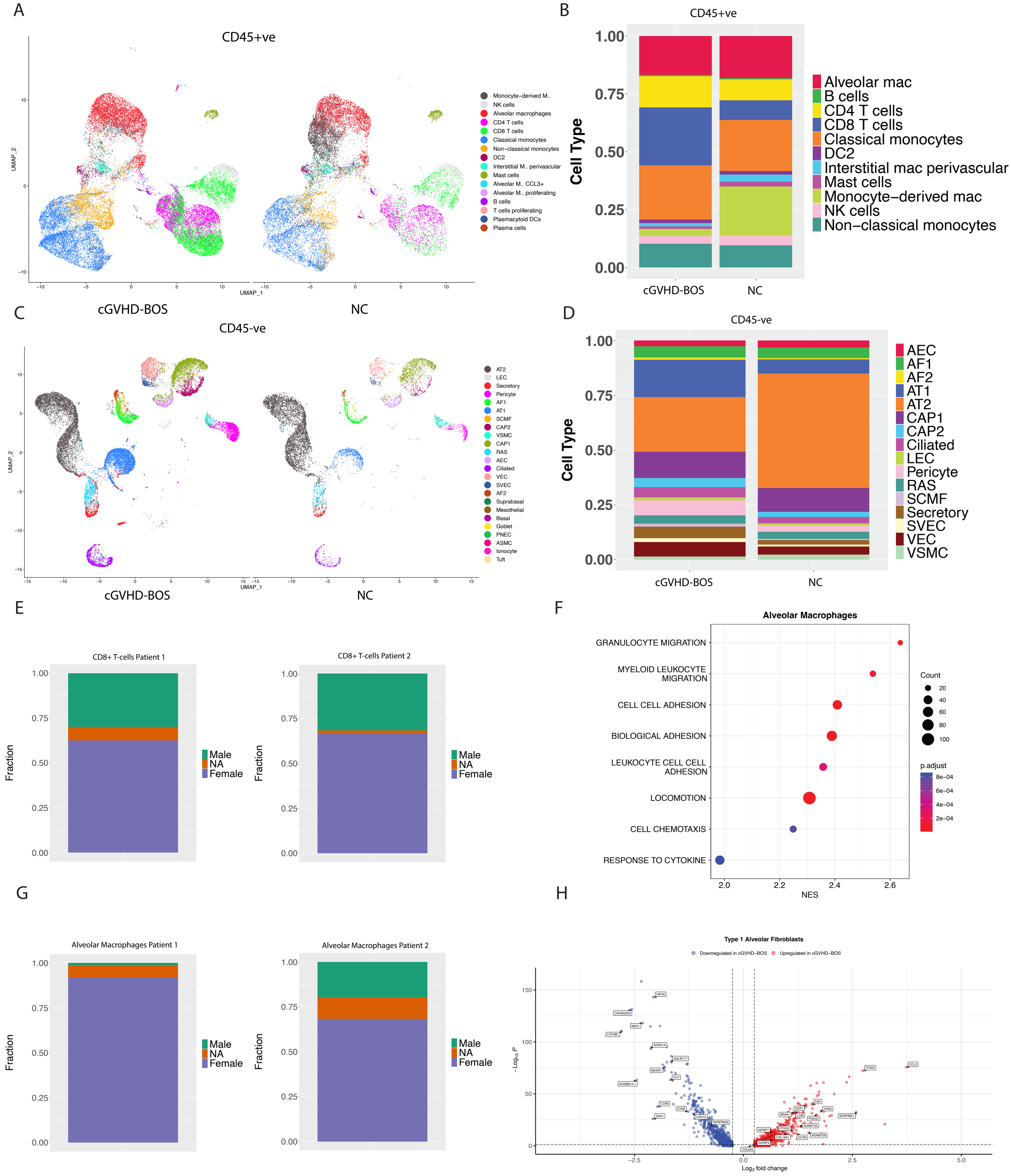

Supplementary Figure S10. (A) UMAP projections for CD45+ve cell types stratified by condition **(B)** Cell type fractions for CD45+ve cell types with > 300 cells stratified by condition **(C)** UMAP projections for CD45-ve cell types stratified by condition **(D)** Cell type fractions for CD45-ve negative cell types with > 300 cells stratified by condition **(E)** Sex of lung-derived CD8+ T-cells in two male cGVHD-BOS patients with female stem cell donors. NA signifies that cell sex could not be determined due to lack of expression of both X and Y associated genes **(F)** Dot plot with enrichment of gene ontology (GO) terms related to macrophage chemotaxis, adhesion, and cytokine response in cGVHD-BOS alveolar macrophages compared to normal controls (NC). P-values for NES were corrected for multiple comparisons using the Benjamini-Hochberg method. NES, normalized enrichment score. **(G)** Sex of lung-derived aMac in two male cGVHD-BOS patients with female stem cell donors **(H)** Volcano plot illustrating expression of pro and anti-fibrotic genes in AF1 in cGVHD-BOS versus NC. Differences in gene expression were quantified using log2fold change and statistically significant differences were identified using Wilcoxon Rank Sum Test and a Bonferroni corrected P-value <0.05

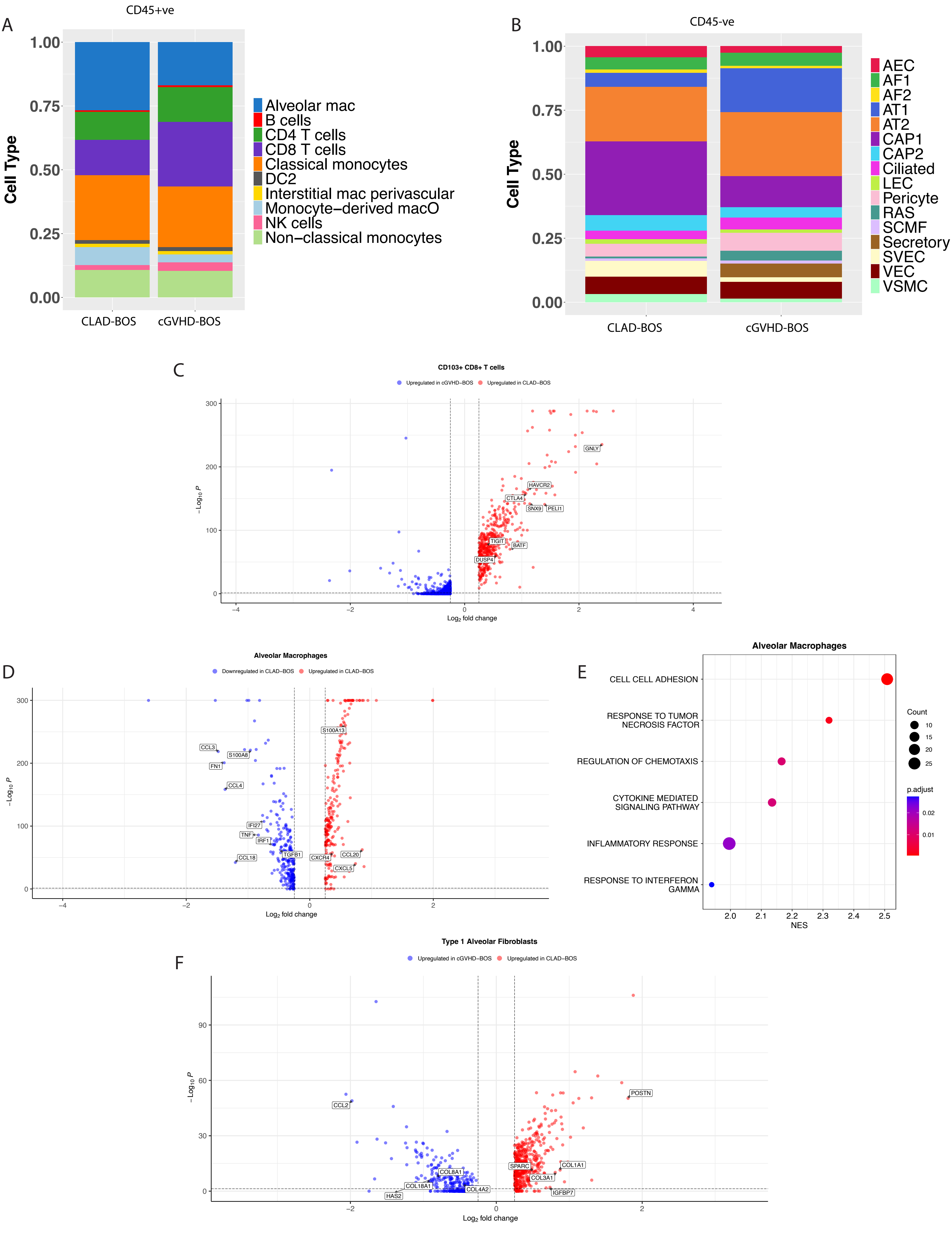

Supplementary Figure S11. (A) Cell type fractions for CD45+ve and **(B)** CD45-ve cell types with > 300 cells stratified by condition **(C)** Volcano plot illustrating differentially expressed genes in CD8+ CD103+ T-cells and **(D)** alveolar macrophages in CLAD-BOS vs cGVHD-BOS. Differences in gene expression were quantified using log2fold change and statistically significant differences were identified using Wilcoxon Rank Sum Test and a Bonferroni corrected P-value <0.05 **(E)** Dot plot with enrichment of gene ontology (GO) in cGVHD-BOS alveolar macrophages compared to CLAD-BOS. P-values for NES were corrected for multiple comparisons using the Benjamini-Hochberg method. NES, normalized enrichment score. **(F)** Volcano plot illustrating differentially expressed genes in AF1 in CLAD-BOS vs cGVHD-BOS.

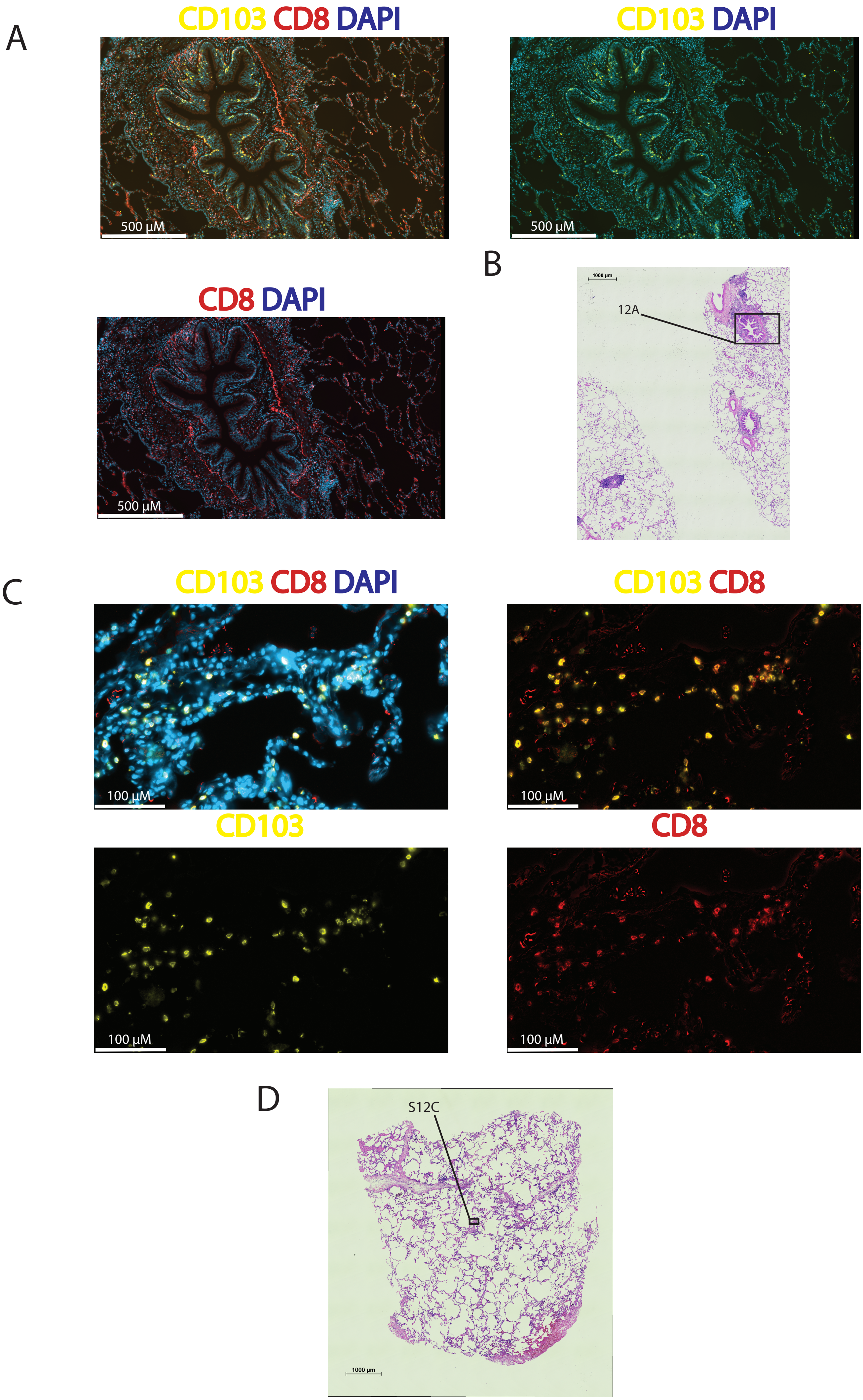

Supplementary Figure S12. (A) mIF images at 20X power illustrating infiltration of a terminal bronchiole by CD8+/CD103+ T-cells in a CLAD-BOS patient and **(B)** H and E image annotated with corresponding location **(C)** mIF images at 20X power illustrating localization of CD8+/CD103+ T-cells to alveolar septa in a cGVHD-BOS patient and **(D)** H and E image with corresponding location. Scale bars (A) 500 µM (B) 1000 µM (C) 100µM (D) 1000 µM

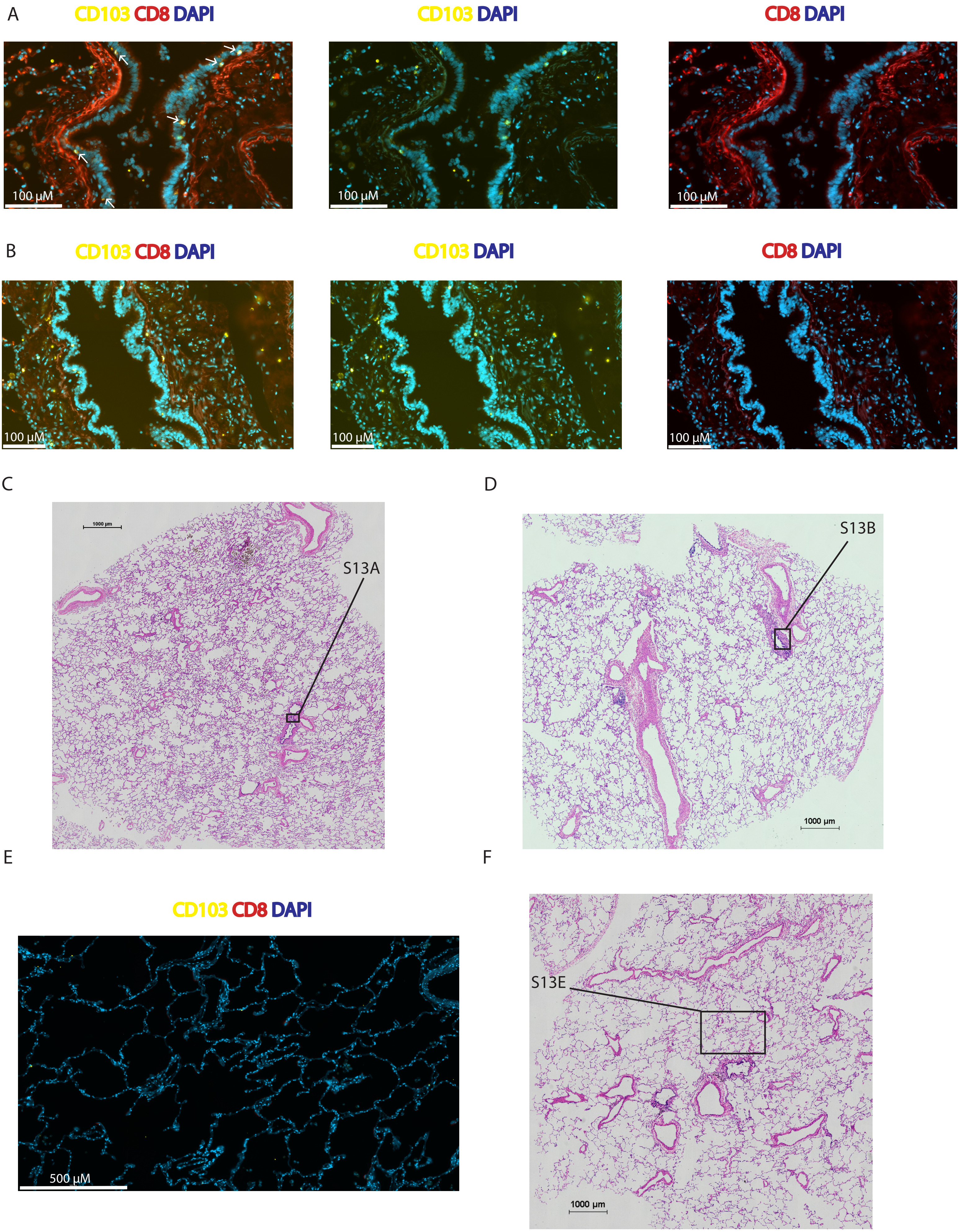

Supplementary Figure S13. (A-B) Multiplex immunofluorescence (mIF) images at 20X power illustrating terminal bronchioles from two different normal control (NC) patients, with DAPI, CD103 and CD8 fluorescent staining and (C-D) H and E images annotated with corresponding locations of mIF images (E) mIF image at 20X of alveolar septa from a third NC patient and (F) H and E annotated with corresponding location. Few CD8+/CD103+ T-cells are highlited with arrows in (A), however they are not clearly identified in (B) or (E). Scale bars (A-B) 100 μM (C-D) 1000 μM (E) 500 μM (F) 1000 μM
